# Predicting rate of penetration (ROP) with machine learning models: A physics-validated comparative case study of Gradient Boosting Regressor (GBR), Random Forest (RF), and K-Nearest Neighbours (KNN)

**DOI:** 10.1371/journal.pone.0360046

**Published:** 2026-11-06

**Authors:** Musaab Magzoub, Noora Al Mansoori, Mohammed Abdalla Ayoub Mohammed, Almostafa Alhadi, Kaushik Sivaramakrishnan, Munawar Shaik, Abu Bakar Adni

**Affiliations:** 1 Department of Chemical and Petroleum Engineering, United Arab Emirates University, Al Ain, United Arab Emirates; 2 Hildebrand Austin Department of Petroleum and Geosystems Engineering, The University of Texas at Austin, Austin, Texas, United States of America; 3 Chemical and Process Engineering, Faculty of Engineering, University of Strathclyde Bahrain, Kingdom of Bahrain; King Mongkut's University of Technology North Bangkok, THAILAND

## Abstract

A successful drilling operation plays a vital role in the profitability of exploration and production of hydrocarbon reserves. However, these operations can come with high drilling rig rental costs. Reliable predictions of Rate of Penetration (ROP) can avoid unnecessary drilling equipment and rig rental expenses. In this study, Gradient Boosting Regressor, Random Forest, and K-Nearest Neighbours were developed to predict rate of penetration based on drilling operational data. The machine learning models were benchmarked with established empirical models, such as the Bingham and Bourgoyne and Young models, which are typically used to predict the rate of penetration. All machine learning models achieved, coefficient of determination (R^2^) > 0.93 and Average Absolute Percentage Error (AAPE) < 3% on the test dataset. Random Forest under Scenario 1 was identified as the most physically consistent model, attaining strong predictive performance with R^2^ = 0.9494 & AAPE = 1.96%, satisfying both the Weight on Bit (WOB) flounder point, and rotary speed optimum criteria used within the industry. These results support the practical use of machine learning-based ROP prediction as a reliable tool for real-time optimization of drilling parameters, offering a pathway to reduce non-productive time and rig costs while preserving the physical interpretability that drilling engineers require for field decision making.

## 1. Introduction

The rate of penetration (ROP) is a fundamental aspect of drilling operations that impacts both time and cost. Drilling wells has always been a crucial process for different applications, not only in oil and gas exploration, but also in geothermal/steam energy extraction, CO_2_ sequestration, hydrogen storage, and enhancing oil production during tertiary recovery [1–3]. According to the U.S. Energy Information Administration in 2016 [4], drilling takes 30%−60% of the average cost per well, which varies from $4.9 MM to $8.3 MM for onshore wells and $120 MM to $230 MM for offshore wells. Given the high daily costs of renting drilling rigs, it is important to optimize ROP to accelerate drilling and reduce overall expenses.

The Rate of Penetration (ROP) is the most pivotal technical metric during drilling processes. ROP signifies the rate at which a drill bit penetrates a formation during drilling operations, usually quantified in units of depth per unit of time [1]. ROP is affected by several parameters, such as weight on bit (WOB), rotary speed (RPM), mud weight (MW), standpipe pressure (SPP) and flow rate [5–7] which are controllable parameters. These inputs can be optimized to improve ROP but also depend on the lithology and geological properties of the deposit being drilled. For example, raising WOB or RPM usually increases ROP, yet there is a limit to this increase before degrading owing to equipment wear or wellbore instability [5,8]. Lithology, which is the composition and strength of the formation being drilled, also has a vital part in ROP. This equation does not apply in hard formation, where the ROP is not exactly proportional to the rotation speed [9,10]. Harder formations require more energy and time while softer formations offer higher drilling rates [11,12]. These interactions need to be incorporated into effective ROP prediction models, balancing the controllable inputs with the geological restrictions to obtain optimal performance [13].

ROP models were first developed by Cunningham [14] in 1960 and have been modified and further researched by Maurer [15], Galle and Woods [16], Bingham [17], Bourgoyne Jr. and Young Jr [18]., and Warren [19]. The classic approaches to ROP prediction include mechanistic and empirical models. Among these models, Bourgoyne and Young (BY) model [18] and Bingham model [17] are the two most commonly used models. The BY model was developed to provide a practical method for estimating ROP by integrating eight drilling parameters, including WOB, RPM, and bit diameter. The semi-empirical model utilizes multiple regression approaches to fit eight coefficients for these variables [20]. The BY and Bingham models are both well-known for their simplicity and success in well planning; however, their reliance on regression techniques restricts their flexibility in accounting for the more complicated, nonlinear connections that typically control ROP during real-time drilling operations.

In recent years, machine learning has emerged as an integral technique for improving the prediction of ROP. Machine learning algorithms like random forest (RF) and gradient boosted regression trees (GBR) are effective at capturing non-linear and complex correlations between drilling parameters and rate of penetration. Unlike traditional models, machine learning approaches do not assume any predefined equations or correlations between the variables. Instead, they analyze huge data sets to detect trends and optimize ROP predictions in real time. Machine learning models have been demonstrated to generally outperform traditional models in prediction accuracy, especially when used in complex and unpredictable drilling situations machine learning models are very suitable for addressing the innate nonlinearity of drilling processes [21,22]. Machine learning models such as random forest, support vector machines, neural networks and KNN have been widely used in areas other than petroleum engineering to predict the mechanical behavior of composite materials and polymer nanocomposites, such as tensile strength, hardness and fracture toughness, showing the broader applicability of these models [23,24].

The machine learning models can incorporate complicated interdependencies of controllable parameters like WOB and RPM and formation-specific elements like lithology and porosity through the analysis of historical data from past drilling operations [25–27]. The capacity of machine learning to react to real time data further increases its utility in modern drilling operations, as circumstances can change rapidly, requiring regular adjustments to drilling parameters to maintain optimal ROP [28].

Huang et al. [1] used Random Forest (RF) to estimate the ROP from drilling operations in southeast Iraq. The model showed an R^2^ of 0.955, MSE of 0.119 and c (AARE) of 7.68%. Then, the model was validated by SHAP and sensitivity analysis which indicated that fracture pressure, kinematic viscosity and rotating speed (RPM) were the most influential characteristics determining ROP. Allawi et al. [29] used drilling parameters and petrophysics data such as gamma ray (GR), density, neutron, calliper and discrete lithology data to estimate ROP in the West Qurna Field, Iraq. They generated a random forest (RF), gradient boosting model (GBM), extreme gradient boosting (XGBoost) and adaptive boosting (AdaBoost) models. Among the four models, the GBM model had the greatest R^2^ value of 0.75.

Sugeno and Mamdani fuzzy inference system (FIS) was used for predicting the weight on bit in Khosravanian et al. [30]. The findings obtained show that the Sugeno type FIS is better than Mamdani type FIS in estimating the weight on the bit. Yavari et al. [31] developed an Adaptive Neuro-fuzzy Inference System (ANFIS) to forecast ROP in different formations in Iran. The model was better than the empirical models such as BY and Hareland model with R^2^ > 0.86 in different formation. The highest R^2^ was obtained in Upper Limestone with R^2^ of 0.9993. The fuzzy network was superior to the empirical models in capturing the complicated nonlinear interactions between the drilling parameters and ROP when compared to the Bourgoyne–Young and Hareland–Rampersad models. Ayoub et al. [9] also used the ANFIS model for ROP prediction. The ANFIS model showed excellent performance with R^2^ of 0.98 and Average Absolute Percent Error (AAPE) of 1.47% compared to Bingham and BY models. Such a greater accuracy was notably visible when the field data was sufficient, establishing ANFIS as the most reliable approach for the ROP prediction in the tested formations.

Recent developments have shifted the application toward deep learning architectures such as Long Short-Term Memory (LSTM) networks, a variant of recurrent neural networks (RNN), are particularly well-suited to ROP prediction because drilling sensor streams are inherently time-series in nature, with ROP at any given depth being influenced by the trajectory of preceding drilling parameters. Ji et al. [32] proposed an optimized LSTM model for ROP prediction, applying feature selection based on Pearson correlation and including 8 crucial inputs, such as formation density, pore pressure and drilling fluid density. The model gave R2, RMSE and MAPE of 0.978, 0.287 and 12.862, respectively. This work established a benchmark for LSTM performance in a single well setting and demonstrated that careful feature pre-selection is as important as network architecture.

Zhang et al. [33] proposed Explainable Hybrid Temporal Graph Neural Network (EHTGNN), which does not rely on specified physical priors to autonomously recognize multivariate interactions and temporal patterns in drilling processes. graph-based deep models combined temporal attention with graph convolutions for explainable prediction. These deep models excel at modelling complex and high-dimensional patterns, achieving a mean absolute error (MAPE) of 16.6% and an R^2^ of 0.994.

Another recent work is the hybrid deep learning architectures that integrate the spatial feature extraction capability of CNNs with the temporal modelling strength of LSTM. Bai et al. [34] proposed the CBT-LSTM model, which integrates a 2D convolutional neural network (2D-CNN) for multi-feature extraction, a bidirectional LSTM (BiLSTM) for capturing bidirectional temporal information, and a temporal pattern attention mechanism to dynamically weight features across time steps. The study focused on the problem of subsurface uncertainty and parameter complexity in real-time ROP evaluation, which has been a persistent challenge. The current innovation in data-driven ROP modelling is the hybrid CNN-LSTM architecture combining local feature extraction (CNN) and long-range sequence modelling (BiLSTM). The model obtained R^2^, MAPE, RMSE and MAE values of 0.9769, 0.0357, 9.3101% and 0.0295 respectively. However, strictly data-driven models often make physically inconsistent predictions when extrapolating outside of the training data settings, a problem that is particularly troublesome in drilling where formation parameters rapidly change across lithological boundaries.

Hybrid physics-machine learning models have been recommended for incorporating domain knowledge directly into the learning framework. Jiao et al. [35] performed a large amount of research on hybrid physics-machine learning (ML) methods to perform ROP prediction in the Halahatang Oil Field (Tarim Basin), China. The first one is residual modelling, the second one is integrated coupling, the third one is simple averaging of physics and machine learning predictions, and the last one is bootstrap aggregating to combine the outputs of multiple physical models as an ensemble. The residual modelling approach was found to show physical plausibility while capturing the nonlinear departures that empirical models fail to represent, achieving an outstanding R^2^ of 0.9936.

The objective of this research is to examine and identify the best data-driven machine learning model for prediction of ROP using operational data from a Sudanese oilfield based on a dataset of 504 data points. In this study, the performance of three different machine learning models (GBR, RF, and KNN) is compared with each other to evaluate the best performing machine learning model and then the final selected machine learning model is compared to the traditional empirical models, in this study, the Bingham model and BY model. This research aims to improve accuracy and operational efficiency of ROP prediction by harnessing the power of machine learning, providing a strong candidate to conventional approaches.

The ROP Modelling covers a wide range of methodologies to forecast and optimize the drilling efficiency. Generally, these methodologies fall into three categories: empirical models, data driven approaches and hybrid models. Empirical models are based on well-known physical principles of drilling dynamics such as Bingham model and BY model, while data-driven methods use statistical and machine learning techniques to forecast ROP from past data. On the other hand, hybrid models combine both approaches to capture the best of both worlds [36].

The advancement of data-driven models, particularly in the last several years, has substantially improved the accuracy of ROP prediction. Machine learning algorithms such as GBR, RF and K-Nearest Neighbours (KNN) have shown great potential in capturing the complicated nonlinear interactions between different drilling parameters and ROP. These models are trained on large datasets, which allow them to learn the complex interactions between factors such as WOB, RPM, and formation characteristics, which are typically difficult to capture with traditional methods [37].

Gradient boosting regression (GBR) is an ensemble learning technique that builds models in a sequence where each successive weak learner (usually a shallow decision tree) is trained to approximate the negative gradient of the loss function with respect to the current ensemble predictions [38]. GBR improves its predictions by minimizing a given loss function, here the mean squared error, in a manner that stresses the correction of the greatest errors. Starting from an initial constant prediction, the algorithm iteratively fits a new tree to the pseudo-residuals of the prior ensemble and incorporates it through the update Eq. (1):


$$\begin{array}{c} {\hat{F}}_{m}(x)={\hat{F}}_{m-\text{1}}(x)+\;\alpha \cdot h_{m}(x) \end{array}$$ (1)

where ${\hat{F}}_{m-\text{1}}(x)$ is the ensemble prediction at iteration m−1, $h_{m}(x)$ is the new tree fitted to the pseudo-residuals, and α∈(0,1) is the learning rate that scales the contribution of each successive tree. For regression tasks, the pseudo-residuals under the mean squared error loss simplify to the ordinary residuals $r_{im}=y_{i}-{\hat{F}}_{m-\text{1}}(x_{i})$, making the iterative correction intuitive: each tree targets the remaining unexplained variance from the prior iteration. The final prediction is the weighted sum of all M individual tree outputs:


$$\begin{array}{c} \hat{F}(x)=\;{\hat{F}}_{\text{0}}(x)+\alpha \cdot \sum_{m=\text{1}}^{M}h_{m}(x) \end{array}$$ (2)

GBR handles complex, nonlinear interactions in high-dimensional data because it executes gradient descent in function space rather than parameter space [38,39]. The maximum tree depth limits the complexity of each individual learner, the learning rate limits the step size at each iteration, and the subsampling ratio, drawing a random fraction of training samples at each iteration without replacement, introduces stochasticity that further reduces variance to control overfitting [38]. The number of estimators (N), learning rate (α), maximum tree depth (D), subsampling ratio, minimum number of samples per leaf, and the minimum number of samples required to split were the six hyperparameters that were improved in this work utilizing Bayesian optimization.

Random Forest (RF) is another ensemble learning method that generates multiple decision trees during training. In contrast to GBR, which constructs trees sequentially, RF produces trees independently and concurrently [40]. Each tree is cultivated on a bootstrap sample of the training data, and at each node division, only a randomly chosen subset of features regulated by the max_features hyperparameter, is evaluated as potential split variables. This dual randomization decouples the different trees, hence significantly diminishing ensemble variance compared to any single tree, while maintaining a low bias [40]. In regression problems, the final Random Forest prediction is the arithmetic mean of all individual tree outputs. Because each tree is exposed to only a subset of the data and features, Random Forest is intrinsically resilient to overfitting and demonstrates consistent performance on high-dimensional datasets with associated predictors. In contrast to GBR, RF does not use a learning rate because trees are not fitted to residuals incrementally. This study primarily tunes the hyperparameters, the number of estimators *N*, the maximum tree depth 𝐷, and the maximum number of features evaluated at each split.

K-Nearest Neighbours (KNN), in contrast to Gradient Boosting Regression (GBR) and Random Forest (RF), is an instance-based learning technique that does not create an explicit model during the training phase [41]. Predictions are generated at query time by identifying the K most analogous training samples to the input point using the Euclidean distance metric. The shortest Euclidean distances between data points and the *k* facts are selected [23]. In regression tasks, the predicted value is the arithmetic mean of the target values of the K nearest neighbours. KNN is occasionally characterized as a ‘lazy learner’ due to its lack of model fitting [42], it postpones all computations until the prediction phase, resulting in rapid training but more sluggish inference as the dataset expands. One practical benefit of KNN is that it is conceptually simple and the predictions are explicitly related to certain training data. KNN is sensitive to irrelevant features and fluctuations in feature size and hence requires constant feature scaling in order to obtain reliable performance [39]. In this work two hyperparameters were optimized using Bayesian optimization. These were the number of neighbours (K) which controls the bias-variance trade-off of the local approximation and the leaf size which determines the threshold at which the KD-tree switches to brute-force search. This affects query efficiency but does not affect the prediction accuracy.

In addition to pure machine learning techniques, hybrid models have become a popular approach for predicting ROP. These models combine physical knowledge of deterministic models with flexibility of data-driven techniques. This allows hybrid models to maintain the interpretability of physics-based models and use machine learning to fine-tune predictions with real-time data. For example, a hybrid model may start with a physics-based estimate of ROP and then improve the estimate using machine learning algorithms that include site-specific geological variations or operational factors [36]. Li et al. [43] optimized the drilling parameters based on a hydraulic-mechanical specific energy. They proposed an innovative sliding window segmentation with the hybrid MSE which enhanced time-series data processing and yielded superior prediction.

The Bingham model is one of the early models developed to predict the ROP during drilling operations. First introduced by M.G. Bingham in 1964 [44], the model was formulated to establish a quantitative relationship between drilling parameters and ROP, focusing primarily on WOB and RPM. The equation is expressed in Eq. (3).


$$\begin{array}{c} ROP=K{\left(\frac{WOB}{d_{b}}\right)}^{a}\cdot N \end{array}$$ (3)

‘WOB’ is the weight on bit, ‘d_b_’ is the bit diameter (inches), ‘K’ is the proportionality constant that accounts for formation strength, ‘N’ is the RPM. The constant ‘a’ represents the rock’s resistance to penetration. It quantifies hardness of t the formation, influencing the overall efficiency of the drilling process [45]. Notice that the values of both ‘K’ and ‘a’ differ across different fields. Therefore, they must be identified empirically by multiple regression and curve fitting techniques. Particle Swarm Optimization (PSO), an advanced optimization technique, was applied to calibrate the ‘K’ and ‘a’ coefficients. Two optimizations were tested to allow a more rigorous and fairer comparison against the machine learning models. The values of ‘K’ and ‘a’ in this study are presented in Table 1:

**Table 1:** The value of ‘K’ and ‘a’ constant in bingham model.

Optimization Technique	K	a
Multiple Regression	0.18	0.38
Particle Swarm Optimization (PSO)	0.1805	0.2018

The equation provides insight into how the ROP changes with variations in the WOB, bit size, and RPM. In this formulation, the term ${\left(\frac{Wob}{d_{b}}\right)}^{a}$ indicates that ROP is influenced by the effective weight per unit area on the bit. Increasing WOB typically results in higher penetration rates because greater force is applied to break the rock [36]. However, this effect is not linear; beyond a certain point, further increases in WOB do not significantly improve ROP due to factors such as bit wear or excessive heat generation. The model, while relatively simple, offers a practical method for estimating ROP based on readily measurable drilling parameters. It assumes a power-law relationship between these variables, making it more adaptable to real-world drilling conditions. However, it does not account for other complex interactions, such as the influence of formation properties, mud type, or downhole vibrations, which can significantly impact the actual ROP during operations [46]. As a result, it is most effective in uniform, stable formations and is less accurate in highly heterogeneous geological environments.

Despite its limitations, the Bingham model has been foundational in drilling engineering. It provides a baseline for understanding the interplay between mechanical forces and drilling performance, and it continues to be used for comparative analysis alongside more advanced models, such as the BY model, which incorporates additional variables and nonlinearities to improve accuracy in more complex drilling scenarios.

The Bourgoyne and Young (BY) model, introduced in 1974 [18], is one of the most widely used empirical models in the petroleum industry for predicting the ROP during drilling which incorporates a total of 8 drilling parameters, including formation strength, depth of formation, effect of formation compaction, weight on bit (WOB), bit wear, bit hydraulics, pressure difference across hole bottom, and rotating speed. Unlike earlier models such as the Bingham model, which primarily considered WOB and RPM, the BY model accounts for a broader range of drilling and formation parameters. The BY model is a robust drilling model applied for roller cone bits [47]. This made it more robust and adaptable to a variety of drilling conditions. The equation in the BY model incorporates eight distinct parameters, as shown from Eqs (4)–(12), where it accounts drilling parameters including formation strength or drillability, depth of formation, effect of formation compaction, bit diameter, weight on bit, pressure differential across hole bottom, rotating speed/RPM effect, bit wear and bit hydraulics.


$$\begin{array}{c} ROP=f_{\text{1}}\;x\;f_{\text{2}}\;x\;f_{\text{3}}\;xf_{\text{4}}\;x\;f_{\text{5}}\;x{\;f}_{\text{6}}\;x\;f_{\text{7}}\;xf_{\text{8}} \end{array}$$ (4)

$$\begin{array}{c} f_{\text{1}}=e^{\text{2}.\text{3}\text{0}\text{3}a_{\text{1}}\;} \end{array}$$ (5)

$$\begin{array}{c} f_{\text{2}}=e^{\text{2}.\text{3}\text{0}\text{3}a_{\text{2}}*(\text{1}\text{0}\text{0}\text{0}\text{0}-D)\;} \end{array}$$ (6)

$$\begin{array}{c} f_{\text{3}}=e^{\text{2}.\text{3}\text{0}\text{3}a_{\text{3}}*D^{\text{0}.\text{6}\text{9}}*(gp-\text{9})\;} \end{array}$$ (7)

$$\begin{array}{c} f_{\text{4}}=e^{\text{2}.\text{3}\text{0}\text{3}a_{\text{4}}*D(gp-ECD)\;} \end{array}$$ (8)

$$\begin{array}{c} f_{\text{5}}={\left[\frac{\left[\frac{w}{d_{b}}\right]-{\left[\frac{w}{d_{b}}\right]}_{t}}{\text{4}-\;{\left[\frac{w}{d_{b}}\right]}_{t}}\right]}^{a_{\text{5}}} \end{array}$$ (9)

$$\begin{array}{c} f_{\text{6}}={\left[\frac{N}{\text{6}\text{0}}\right]}^{a_{\text{6}}} \end{array}$$ (10)

$$\begin{array}{c} f_{\text{7}}=e^{-a_{\text{7}}*h\;} \end{array}$$ (11)

$$\begin{array}{c} f_{\text{8}}={\left[\frac{F_{j}}{\text{1}\text{0}\text{0}\text{0}}\right]}^{a_{\text{8}}} \end{array}$$ (12)

Where ‘D’ is the true vertical depth, ‘Gp’ is pore pressure gradient, ‘ECD’ is equivalent circulating density of the drilling fluid, $\left[\frac{w}{d_{b}}\right]$ is ratio of weight on bit to diameter, ${\left[\frac{w}{d_{b}}\right]}_{t}$ is ratio of bit threshold to the bit diameter, ‘N’ is the rotation speed, ‘H’ is the fractional tooth dullness, and ‘F_j_’ is the jet impact factor. The coefficients a_1_ through a_8_ represent empirical constants that are determined through different methods from, multiple regression technique and Particle Swarm Optimization (PSO). The constants a1-a8 are not universal constants, as they vary across different fields and optimization techniques used. Advance optimization techniques such as Particle Swarm Optimization were implemented to provide fair comparison to the machine learning models. The values of a1 – a8 constants for this study are shown in Table 2.

**Table 2:** The values of the constant a1-a8 in bourgoyne and young model.

Optimization Technique	a1	a2	a3	a4	a5	a6	a7	a8
Multiple Regression	1.4	7.5E-05	1.00E-06	1.00E-06	0.65	0.65	0.3	0.3
Particle Swarm Optimization (PSO)	1.16	6.47E-07	1.78E-07	2.01E-05	0.30	1.36	1	1.85E-04

The model is considered an advancement over earlier approaches as it integrates a more comprehensive set of factors that impact drilling. While earlier models like Bingham’s focused on a limited number of variables, the BY model allows for more nuanced predictions by considering additional geological and operational conditions. This makes it more accurate across a wider variety of formations and drilling environments.

Despite its robustness, the BY model still has some limitations. It relies on empirical data; thus, its accuracy is highly dependent on the quality of the input data and the calibration performed for specific formations. Additionally, the model assumes that the relationships between parameters are log-linear, which may not fully capture highly nonlinear or dynamic conditions encountered in some drilling operations.

The investigated well in this study is located in the Muglad Basin, an intracontinental rift basin in Sudan and the largest oil producing basin in the country. The drilled stratigraphic sequence from the surface to the total depth of the well includes seven formations as shown in Table 3:

**Table 3:** Formations description during drilling operation.

Formation Name	Depth Interval, ft	Mineralogy
Adok and Nayil	0–288	shale
Amal	288–486 ft	sandstone
Baraka	487–668 ft	interbedded shale/sandstone
Ghazal	669–728 ft	interbedded shale/sandstone
Zarqa	729–829 ft	interbedded shale/sandstone
Aradeiba	830–1258 ft	interbedded shale/sandstone
Bentiu	1259–1660 ft	sandstone with shale interbeds

The Bentiu Formation encountered at the deepest part of the well is well known as the principal reservoir for oil and gas in the Muglad Basin [48]. Core based petrophysical studies from the northeastern Muglad Basin report that the coarse to medium grained Bentiu sandstone facies have porosity values between 19.6% and 32.0% and permeability values between 1825.6 mD and 8358.0 mD, whereas the finer grained, clay-rich facies have significantly poorer reservoir quality with porosity values between 1.0% and 6.0% and permeability values between 2.5 mD and 10.0 mD [48].

## 2. Methodology

This section outlines the processes followed to prepare the data, perform statistical analysis, and apply machine learning techniques. It includes the steps taken for data cleaning, feature engineering, and the reasoning behind the selection of key features. Additionally, it describes the statistical analyses used to assess data characteristics, multicollinearity, and relationships between features. These techniques are applied using programming language Python in Jupyter Notebook and its extensive libraries for data analysis and machine learning namely, pandas, numpy, scikit learn, matplotlib and Bayes opt. The proposed models were evaluated across three scenarios, each defined by a progressively reduced set of input parameters as summarized in Table 4.

**Table 4:** The proposed scenarios for the ML models.

	Depth	RPMavg	WOBavg	Flow	MWavg	Torque
Scenario 1	✓	✓	✓	✓	✓	✓
Scenario 2	✓	✓	✓	✓	✓	X
Scenario 3	✓	✓	✓	✓	X	X

The use of machine learning approaches on distinct sets of features allows a more extensive comparison and a better understanding of the impact of each feature in terms of whether adding them improves or worsens the predicted accuracy of the model. The workflow chart of this work is presented in Fig 1.

**Fig 1:**
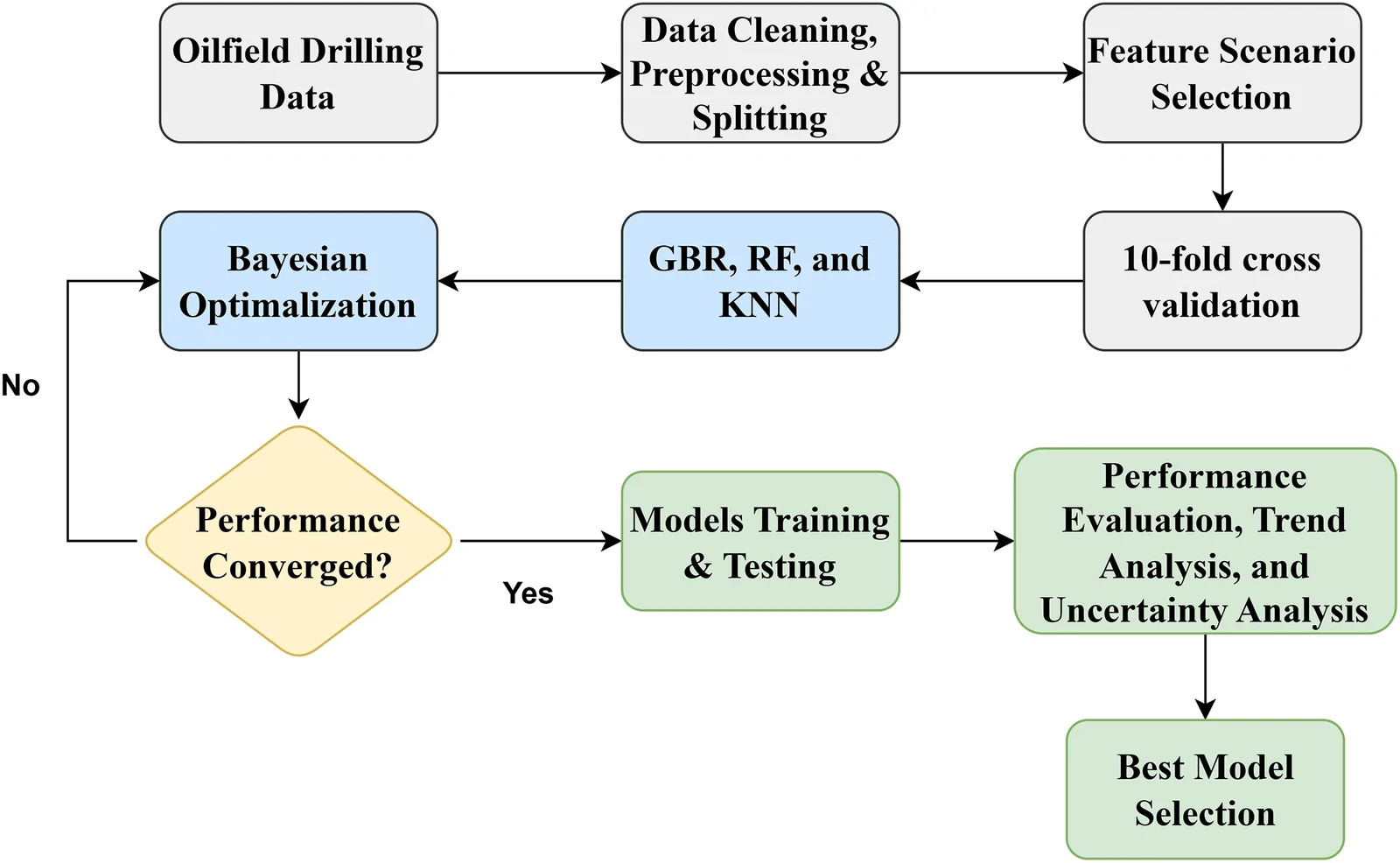
Schematic workflow of the present study. The proposed machine learning models contain three scenarios with different input variations.

### 2.1. Data preparation

The dataset contains 504 data points that were obtained from drilling operations. Each record consists of measurements of depth, rotational speed, weight on bit, mud weight, torque and flow rate, along with the target variable, rate of penetration (ROP), as shown in Table 5. To reduce redundancy while maintaining the significant magnitude of each variable, rotational speed, weight on bit, and mud weight were initially recorded as minimum and maximum values per interval. These were then averaged (RPM_avg_, WOB_avg_, and MWavg) to provide a single representative value per parameter.

**Table 5:** Model input features and target variable.

Variable	Description	Unit
Depth	Measured depth of the well	ft
RPMavg	Averaged rotational speed of the drill bit	Rpm
WOB_avg	Averaged weight on bit	klbf
MWavg	Averaged mud weight	lbm/gal
Torque	Rotational force applied to the drill string	ft-lbf
Flow	Drilling fluid circulation rate	gpm
ROP	Rate of penetration (target variable)	ft/hr

These six parameters were selected as model inputs based on their established physical significance to drilling efficiency and preliminary spearman correlation and variance inflation factor (VIF) screening, which confirmed acceptable multicollinearity among the selected features. The dataset was checked for missing values and divided into training (80%) and test (20%) subsets using a fixed random seed to ensure reproducibility before model development.

The six input features used in this are study, depth, WOB_avg_, RPM_avg_, MW_avg_, torque and flow were selected based on the known mechanistic roles in controlling ROP, as described in classical and contemporary drilling engineering literature. Depth is a basic input in most empirical and data-driven ROP models since it determines the formation compaction and overburden stress that the bit experiences, both of which affect rock strength and drillability as the well depth increases [18]. WOB_avg (weight on bit) is one of the two main controllable drilling parameters and directly controls the mechanical load imposed on the rock face. It is established that ROP improves with WOB to a threshold (the floundering point) beyond which additional bit weight results in diminishing or negative returns due to overloading of the cutting structure [17], [18].

The second primary controllable parameter is the rotary speed, RPMavg, which determines the frequency of the bit-rock contact cycles. ROP usually increases with rotary speed, until an optimal value is reached, when it levels off or decreases because of mechanical constraints, such as bit wear and vibration [17,18]. The mud weight (MW_avg_) affects ROP mainly due to its effect on bottomhole differential pressure. If the mud weight is higher, the hydrostatic pressure against the formation increases, impairing the release of drilled cuttings from the rock face and suppressing the penetration rate [18,49].

Torque is a measure of resistance at the bit-rock interface during rotation and is mechanically coupled to WOB and RPM. It was kept as an input because the torque response has been shown to contain additional information related to formation hardness and bit wear that is not always fully captured by WOB and RPM alone [46]. Its potential redundancy relative to WOB and RPM was then evaluated by the feature-reduction analysis of the scenarios 2 and 3. Flow (mud flow rate) controls bit hydraulics and the efficiency of hole cleaning at the bottomhole. The absence of flow can result in an incomplete removal of the drilled cuttings, thus promoting their re-grinding at the bit face and indirectly reducing the ROP while the other mechanical parameters are still favorable. This hydraulics-related effect is explicitly taken to be considered, as one of the eight governing functions, in the Bourgoyne-Young formulation [18].

### 2.2. Validation strategy

The machine learning models were validated using a holdout validation strategy, in which the dataset was randomly partitioned into training and tested subsets. To assess the model stability, this partitioning was repeated across four train:test split ratios, 60:40, 70:30, 80:20, and 85:15, with 10 independent random seeds evaluated per ratio. The resulting R^2^ and RMSE values across all splits are presented in supporting information. For this study, we decided to proceed with 80:20 train and test ratio.

Once the data split is completed, the next step is data preprocessing which includes normalization. To avoid data leaking, StandardScaler() normalization was fitted only on the training set and then used to modify the test set. The StandardScaler() normalization formula is shown in Eq. 13. Bayesian optimization was employed to tune each model’s hyperparameters with the 10-fold cross-validation R^2^ on the training set, hence tuning decisions were guided by generalization performance, not solely by training fit.


$$\begin{array}{c} x_{scaled}=\frac{x_{Original}-\mu }{\sigma } \end{array}$$ (13)

Where:

- X_Scaled_: the normalized value.
- X: the input value.
- µ: mean.
- σ: standard deviation

The optimized models were then tested on the unseen holdout test set using three error metrics: the coefficient of determination (R^2^), root mean square error (RMSE), and average absolute percentage error (AAPE). The two measures together measure model explained variance and magnitude of prediction error in physically interpretable units. Overfitting diagnostic was conducted by comparing the RMSE gap between training and testing for all models, which confirmed that the regularization limitations enforced through the Bayesian optimization search space effectively prevented memorizing of the training data. The summary of the dataset used to develop the model is shown in Table 6.

**Table 6:** Descriptive statistics of key features and target variable.

	Depth (m)	RPM_avg_	WOB_avg_ (Ton)	MW_avg_	Torque	Flow (GPM)	ROP (m/hr)
Maximum	1660	150	15	11.25	5.56	800	47.7
Minimum	0	100	3	8.5	0.12	419	3.08
Average	830	141.33	9.96	10.21	2.24	638.43	15.64
STD	485.42	10.63	1.50	0.84	1.05	79.58	7.95
Range	1660	50	12	2.75	5.44	381	44.62
Mode	0	150	11	10.8	3	650	14
Skewness	3.55E-18	−1.99	−2.11	−0.44	0.05	−0.14	1.91
Kurtosis	−1.20	5.20	8.88	−1.45	−0.57	−0.40	3.99

### 2.3. Spearman correlation

Spearman’s rank correlation was computed for each scenario to examine the monotonic relationships among input features and the target variable. The analysis was repeated for each scenario as the feature set was progressively reduced. The Spearman correlation for each scenario was illustrated in Figs 2–4.

**Fig 2:**
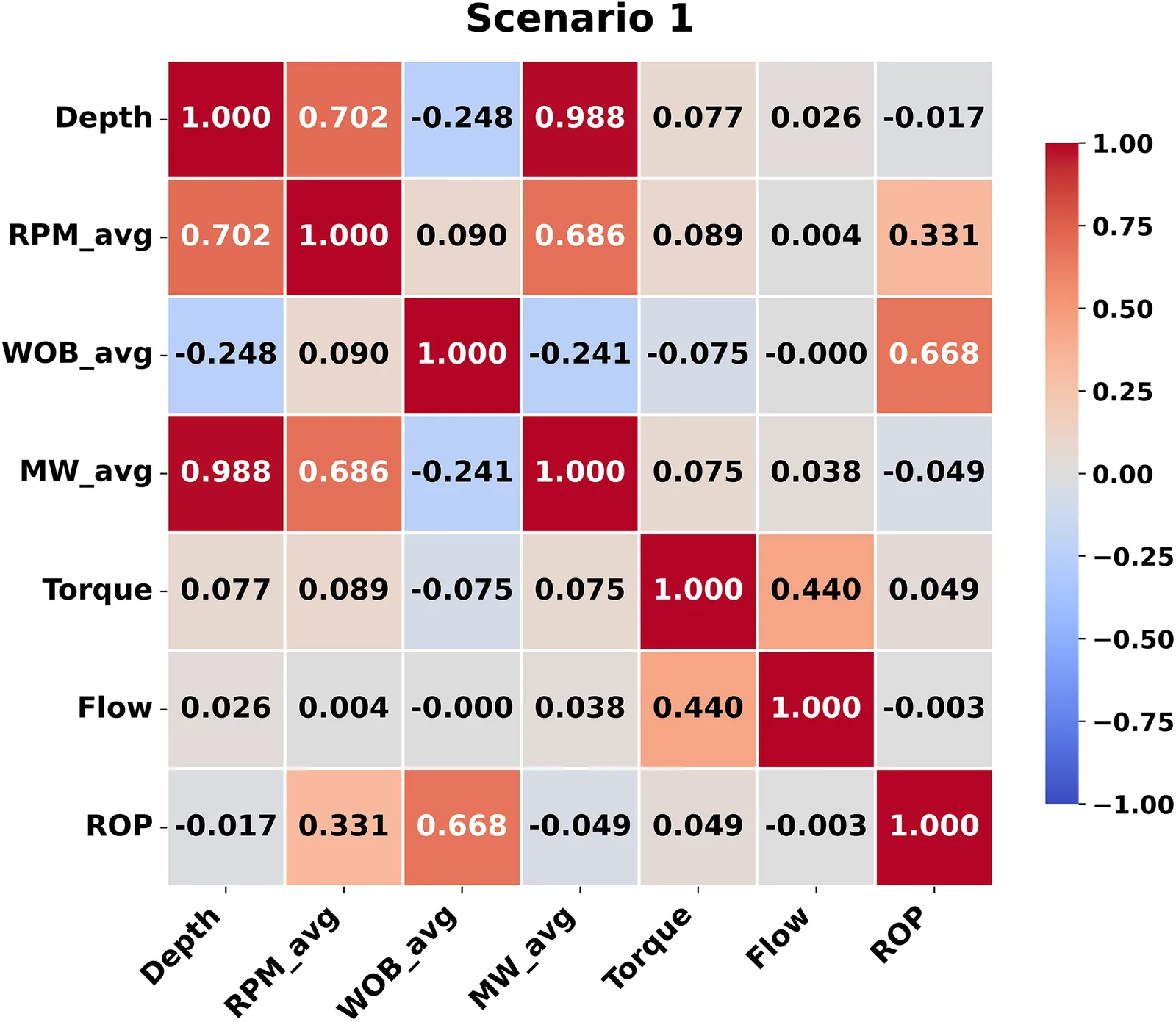
Heat map spearman correlation for scenario 1. Spearman rank correlation heatmap for Scenario 1 (depth, RPM, WOB, MW, torque and flow rate) against ROP. WOB (ρ = 0.668) and RPM ρ = 0.331) show the strongest monotonic relationships with ROP.

**Fig 3:**
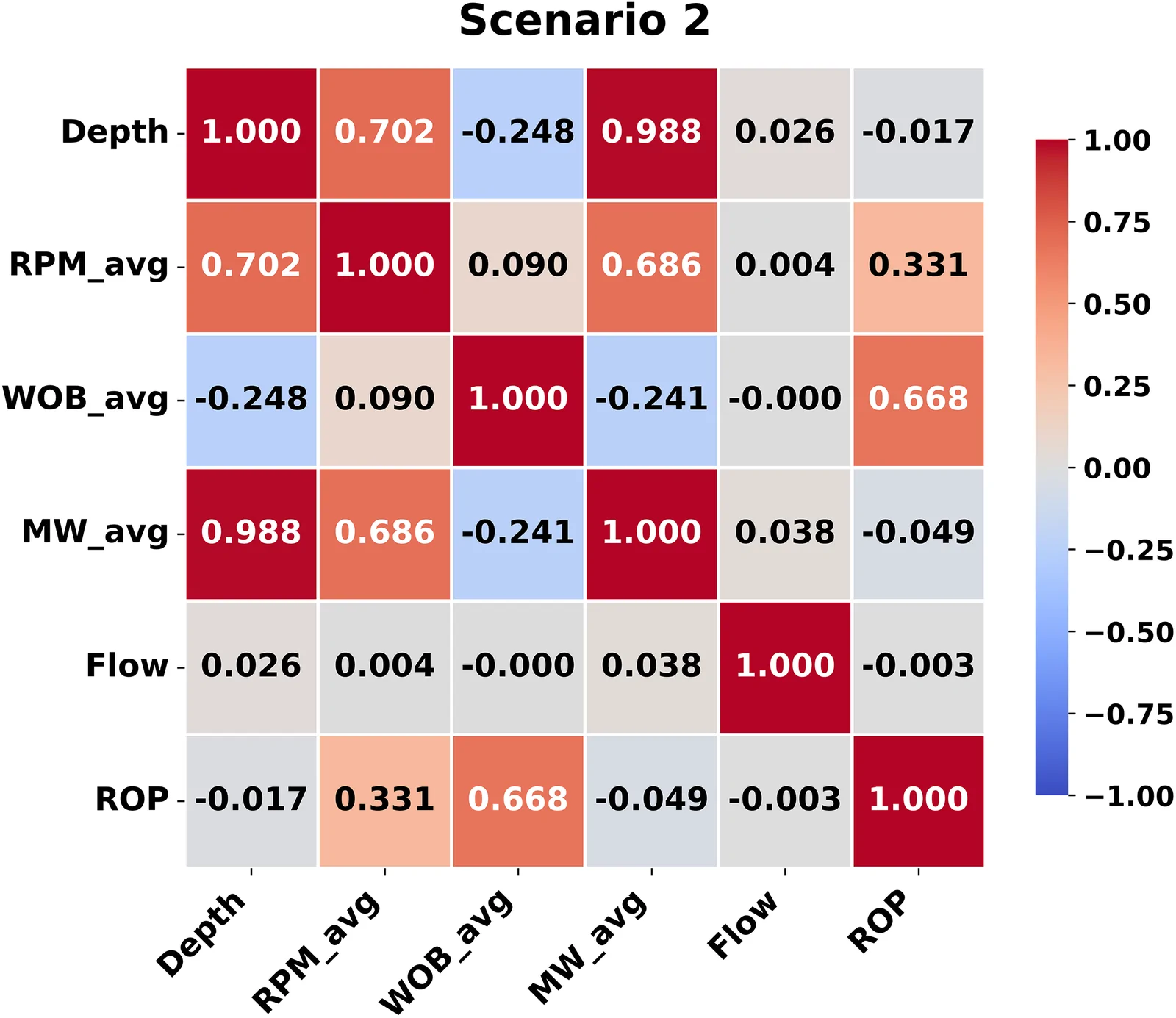
Heat map spearman correlation for scenario 2. Spearman rank correlation heatmap for Scenario 2, with torque removed (depth, RPM, WOB, MW and flow rate) against ROP.

**Fig 4:**
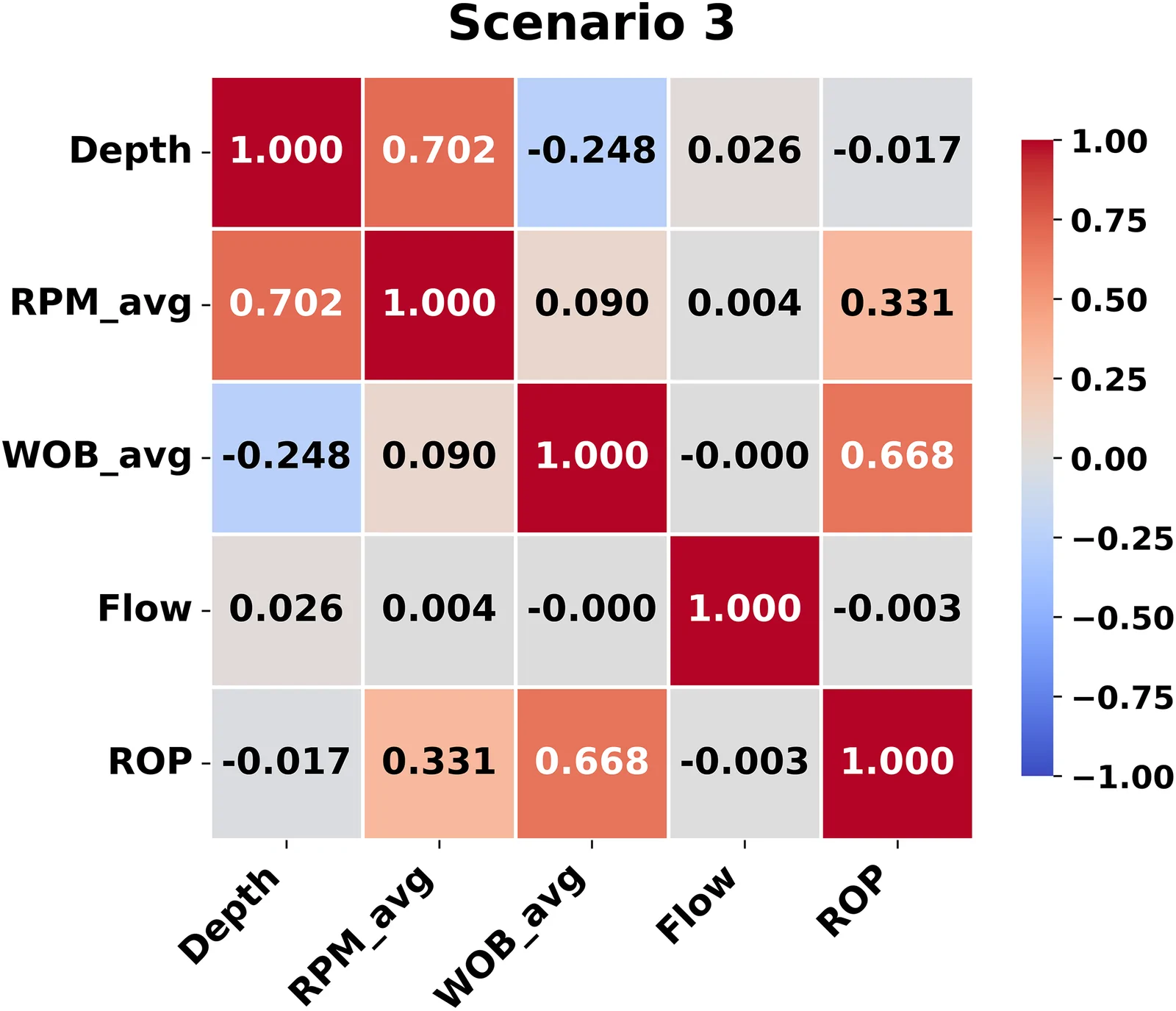
Heat map spearman correlation for scenario 3. Spearman rank correlation heatmap for Scenario 3, with torque and MW removed (depth, RPM, WOB and flow rate) against ROP.

In scenario 1, two pairs of features exhibit strong collinearity, depth-MW_avg_ has r = 0.988 and depth-RPM_avg_ has r = 0.702, indicating that mud weight and rotary speed increase systematically with depth in this drilling program. Torque and flow show moderate mutual correlation of r = 0.440. With respect to ROP, WOB_avg_ shows the strongest positive association, followed by RPM_avg_, while depth and MW_avg_ show weak negative correlations with r = −0.017 and −0.049, respectively. Torque and flow contribute minimally to ROP.

In Scenario 2, the feature-ROP correlations for the remaining variables are unchanged, confirming that torque’s removal does not redistribute signal among other features. The depth-MW_avg_ collinearity remains the dominant inter-feature relationship, causing its further treatment in Scenario 3. The collinearity structure for scenario 3 is considerably simpler. The other significant inter-feature relationship is depth-RPM_avg_ with r = 0.702, which is indicative of the design of the drilling operation rather than physical redundancy. WOB_avg_ still has a substantial correlation with ROP with r = 0.668 and RPM_avg_ has r = 0.331. These two features are the largest contributors to ROP in this limited feature set. Table 7 shows the VIF values for each characteristic in the three scenarios. The VIF estimates the increase in the variance of a given feature due to its linear relationships with the other features in the set. The appropriate value of VIF is under 5, a number between 5 and 10 shows significant collinearity that needs to be solved; a value beyond 10 shows severe redundancy [50].

**Table 7:** Variance Inflation Factor (VIF) across three scenarios.

Feature	Depth	RPM_avg_	WOB_avg_	MW_avg_	Torque	Flow
Scenario 1						
VIF	9.895	3.192	1.915	8.401	1.214	1.237
Scenario 2						
VIF	9.754	3.191	1.913	8.306	–	1.038
Scenario 3						
VIF	2.197	3.178	1.859	–	–	1.010

For scenario 1, the greatest VIF values are for depth and MW_avg_ (9.895 and 8.401, respectively), which are close to the crucial value of 10. This is consistent with the spearman correlation heatmap where the depth and MW_avg_ have almost perfect rank correlation, the greatest pairwise connection seen throughout the complete feature set. This near-perfect correlation reflects the design of the drilling operation of the oilfield. As the mud weight was progressively increased with depth to preserve wellbore pressure integrity, the two variables are practically linearly dependent. The rest of the features RPM_avg_ (3.192), WOB_avg_ (1.915), torque (1.214) and flow (1.237) are also significantly below the threshold of 5 confirming their independence.

In scenario 2, torque is removed. The VIF values of the rest of features are negligibly affected, depth reduces slightly from 9.895 to 9.754, and MW_avg_ decreases from 8.401 to 8.306. This minor adjustment shows that torque was not part of the collinearity problem. The spearman heatmap for scenario 1 indicates a moderate connection between torque and flow with r = 0.440 and nearly zero correlations between torque and all other features. This explains why the collinearity structure does not change when torque is removed.

The critical improvement occurs in scenario 3, where MW_avg_ is removed. The VIF of depth declines significantly from 9.75 to 2.19, and all other features have VIF values less than 4. This confirmed that the main driver for higher VIF in depth was MW_avg_. This conclusion was fully confirmed by the spearman heatmap, which highlighted depth-MW_avg_ collinearity with r = 0.988 as the most important inter-feature association. The physical relevance of depth as a proxy for formation compaction and overburden pressure is valid without redundancy of information already captured by another characteristic, in the absence of MW_avg_.

## 3. Results and discussion

### 3.1. Bayesian optimization of machine learning techniques for ROP prediction

Bayesian optimization is employed as a robust method for hyperparameter tuning of the three machine learning models since it effectively explores the hyperparameter space. In contrast to grid or random search, Bayesian optimization creates a surrogate model to estimate the influence of different hyperparameter configurations on the model performance, allowing it to focus on the most promising areas of the search space [51]. This prevents needless exploration of less effective hyperparameters and converges to the optimal set faster. This verifies that the chosen hyperparameter settings are suitable for improving model accuracy and reducing prediction error, resulting in more reliable and accurate ROP forecasts. The hyperparameter search of each model is shown in Table 8.

**Table 8:** Hyperparameter searches for all models.

Model	Parameter	Bounds
GBR	n_estimators	50–300
	learning_rate	0.01–0.2
	max_depth	3–7
	subsample	0.5–0.8
	min_samples_leaf	5–30
	min_samples_split	5–30
RF	n_estimators	50–400
	max_depth	2–15
	min_samples_leaf	3–20
	min_samples_split	5–20
KNN	n_neighbors	3–50
	leaf_size	2–50

#### 3.1.1. Scenario 1.

Table 9 presents the results of optimized hyperparameters three machine learning models using all six features in the machine learning model. The GBR model achieved the highest testing accuracy with an R^2^ of 0.9858 and AAPE of 1.17%, indicating a strong ability to explain the variance in ROP. The corresponding testing Root Mean Squared Error (RMSE) for GBR was 0.5048, indicating relatively low predictive error. In contrast, the RF model had a slightly lower testing accuracy, with an R^2^ of 0.9494 and AAPE of 1.96%. The KNN model performed slightly worse, with a testing R^2^ of 0.9363 and AAPE of 2.25% indicating moderate predictive accuracy.

**Table 9:** Best hyperparameters in scenario 1.

Model	Parameter	Optimal Value
GBR	n_estimators	106
	learning_rate	0.2
	max_depth	7
	subsample	0.8
	min_samples_leaf	5
	min_samples_split	10
RF	n_estimators	62
	max_depth	15
	min_samples_leaf	3
	min_samples_split	5
KNN	n_neighbors	4
	leaf_size	15

Figs 5–7 present the cross plot of scenario 1 across the machine learning models predicted versus actual values of ROP for the best-performing model, with all six features included in the modeling. The model demonstrates a high level of accuracy, as shown by the R^2^ value of 0.9858 for the line of best fit. The points align closely with the line of best fit, which suggests strong agreement between the predicted and actual ROP values. The KNN and RF models also reached high R^2^ values with a few points scattered away from the perfect line. Table 10 and 11 summarize the results for scenario 1 on the training and testing datasets.

**Fig 5:**
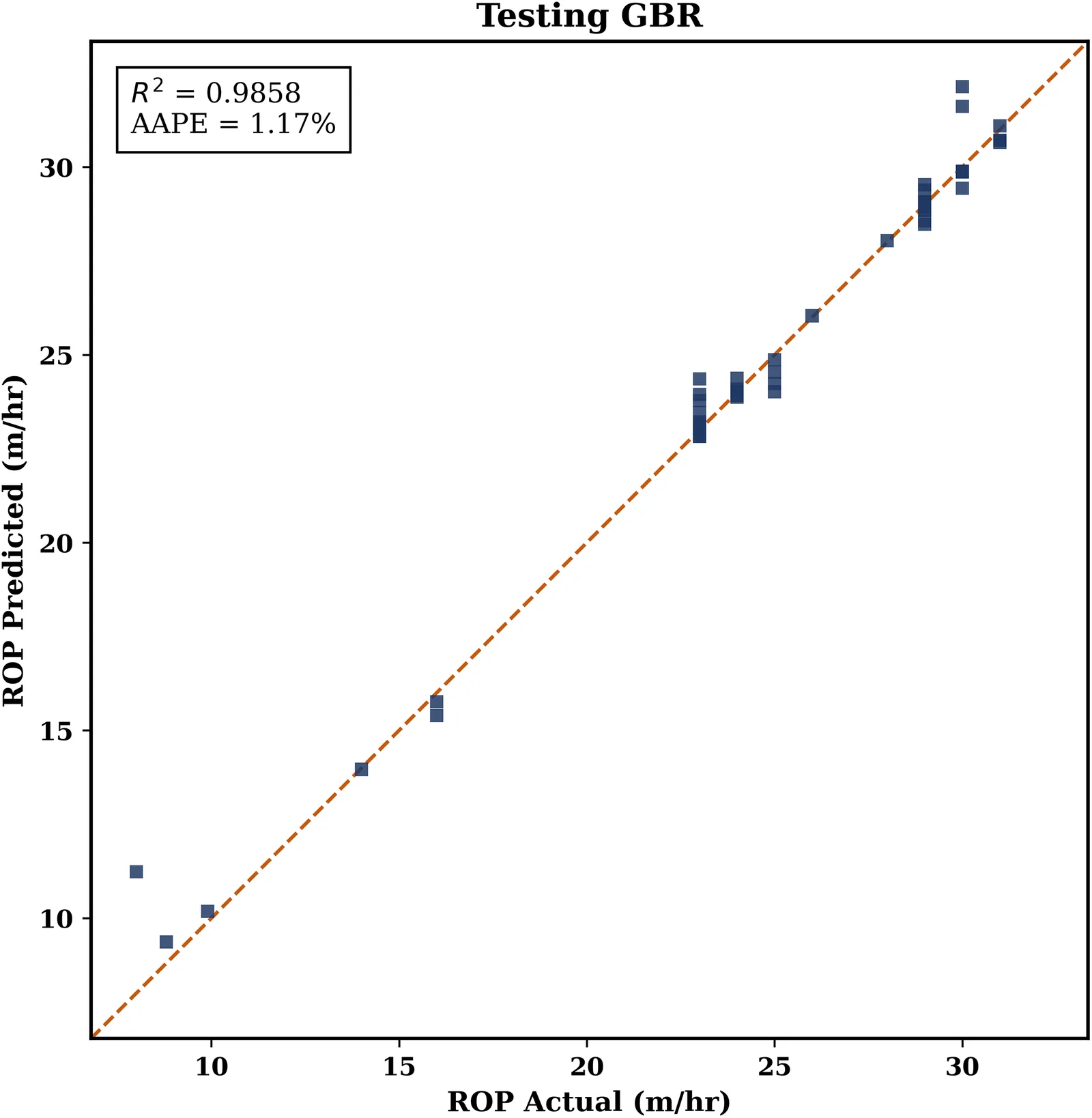
Cross-plots of GBR model predictions for the testing dataset in scenario 1. Predicted versus actual ROP for the Gradient Boosting Regressor (GBR) on the testing dataset with R^2^ = 0.9858 and AAPE = 1.17%.

**Fig 6:**
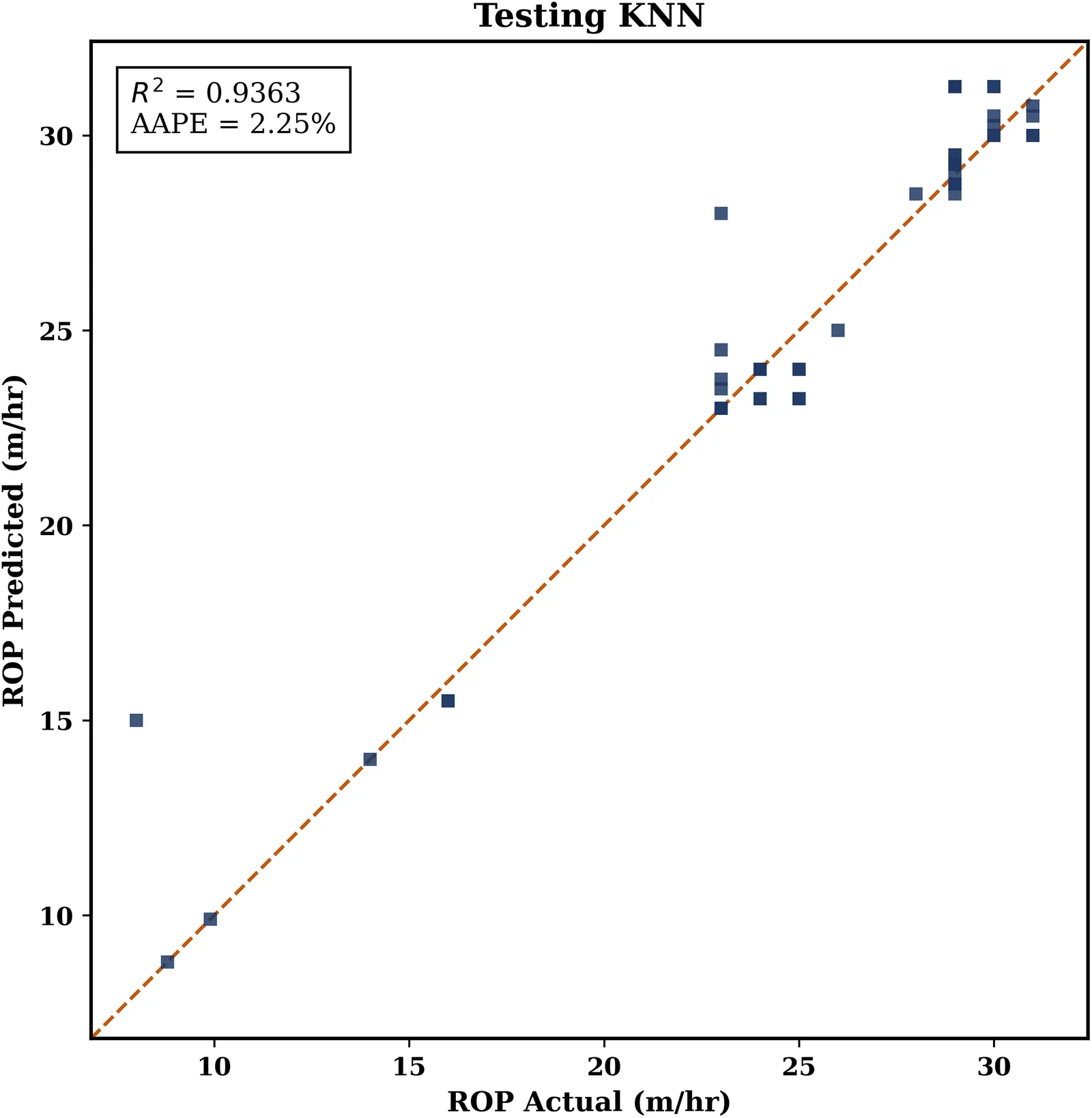
Cross-plots of KNN model predictions for the testing dataset in scenario 1. Predicted versus actual ROP for the K-Nearest Neighbors (KNN) model on the testing dataset with R^2^ = 0.9363 and AAPE = 2.25%.

**Fig 7:**
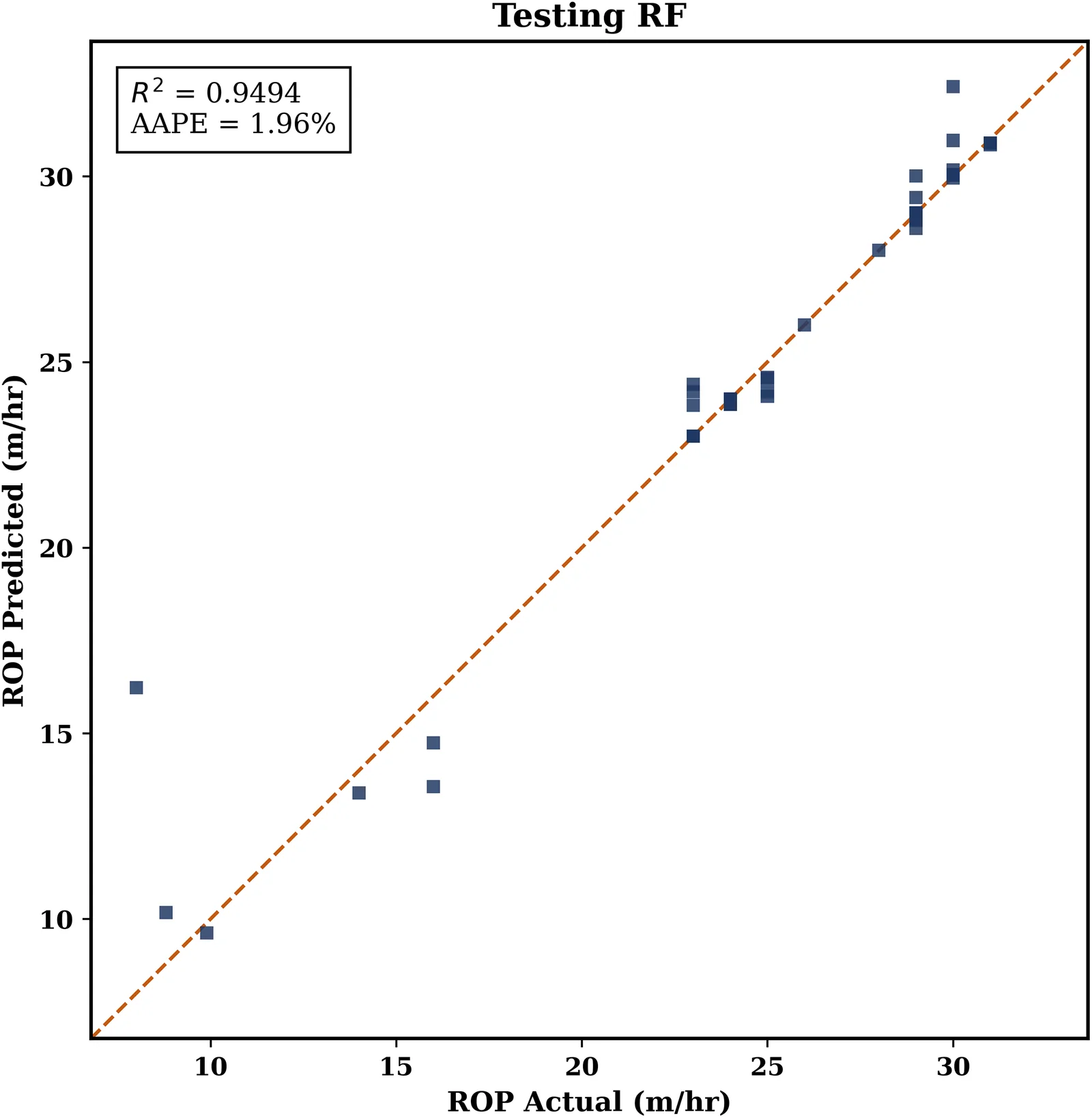
Cross-plots of RF model predictions for the testing dataset in scenario 1. Predicted versus actual ROP for the Random Forest (RF) model on the testing dataset with R^2^ = 0.9494 and AAPE = 1.96%.

**Table 10:** Statistical analyses for training dataset (Scenario 1).

Model	R^2^	RMSE	AAPE, %
GBR	0.9980	0.209	0.52
RF	0.9840	0.934	1.94
KNN	0.9603	0.592	1.13

**Table 11:** Statistical analyses for testing dataset (Scenario 1).

Model	R^2^	RMSE	AAPE, %
GBR	0.9858	0.5048	1.17
RF	0.9494	0.9536	1.96
KNN	0.9363	1.069	2.25

The enhanced performance of GBR compared to RF and KNN with the six features retained is aligned with recent ROP literature. Yehia et al. (2024) [52] similarly found gradient-boosting and random-forest ensembles forming the top-performing cluster among ten algorithms tested on the FORGE geothermal dataset, while KNN lagged. The superiority of GBR in this case is likely due to the advantage of systematic Bayesian hyperparameter optimization (Table 11), which allows the sequential error-correction mechanism of boosting to outperform the averaging-based approach of RF, as also reported by Zhang et al. (2025) [53] for Iraqi field data. The lower accuracy of KNN is consistent with its known vulnerability to correlated, high-dimensional feature spaces where distance-based prediction deteriorates as redundant inputs disrupt the neighborhood structure [54].

#### 3.1.2. Scenario 2.

Table 12 presents the results of optimized hyperparameters with torque removed from the modeling for 3 machine learning models. Compared to the results from bayesian optimization with all six features, GBR remains the best-performing model, but its testing R^2^ has decreased slightly to 0.9779, while the testing RMSE has improved to 0.617, indicating higher accuracy and lower error even after removing torque. This suggests that the removal of torque improved the predictive power of KNN. The Table 13 and 14 summarize the results for scenario 2 on the training and testing datasets.

**Table 12:** Best hyperparameters in scenario 2.

Model	Parameter	Optimal Value
GBR	n_estimators	290
	learning_rate	0.144675
	max_depth	7
	subsample	0.506933
	min_samples_leaf	6
	min_samples_split	21
RF	n_estimators	68
	max_depth	15
	min_samples_leaf	3
	min_samples_split	5
KNN	n_neighbors	3
	leaf_size	10

**Table 13:** Statistical analyses for training dataset (scenario 2).

Model	R^2^	RMSE	AAPE, %
GBR	0.9959	0.3	0.71
RF	0.9821	0.626	1.27
KNN	0.9803	0.659	1.16

**Table 14:** Statistical analyses for testing dataset (scenario 2).

Model	R^2^	RMSE	AAPE, %
GBR	0.9779	0.617	1.45
RF	0.9470	0.975	2.02
KNN	0.9616	0.831	1.55

In addition, RF model’s performance has seen a decrease in testing R^2^, decreasing to 0.947%, and an increase in testing RMSE. KNN outperforms its previous model, with a testing R^2^ of 0.9616. This suggests that the removal of torque improved the predictive power of KNN. Table 14 shows a summary of scenario 2 on testing dataset. Figs 8–10 show the cross plot across the models for scenario 2.

**Fig 8:**
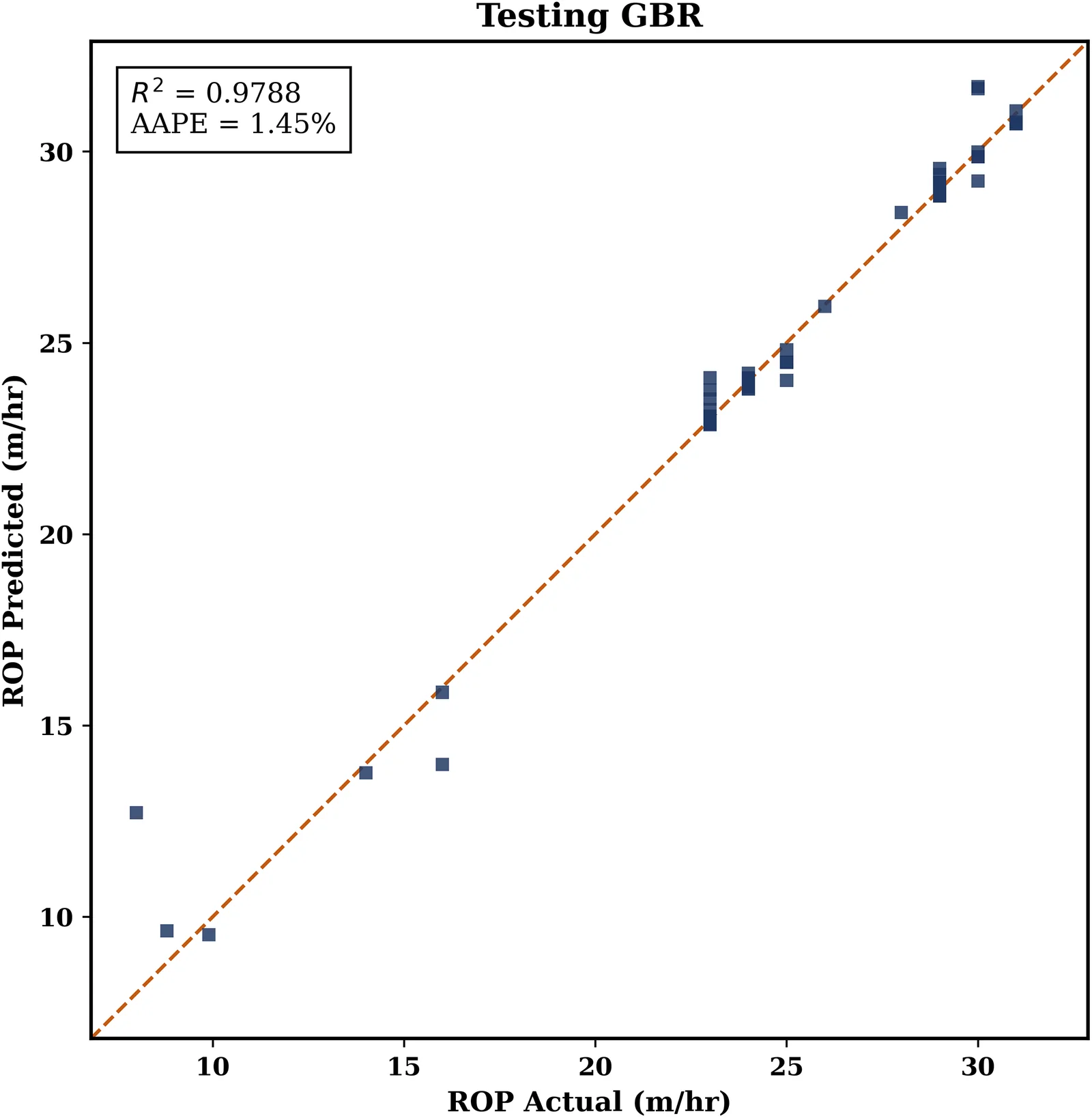
Cross-plots of GBR model predictions for the testing dataset in scenario 2. Predicted versus actual ROP for GBR on the testing dataset, Scenario 2 with R^2^ = 0.9788 and AAPE = 1.45%.

**Fig 9:**
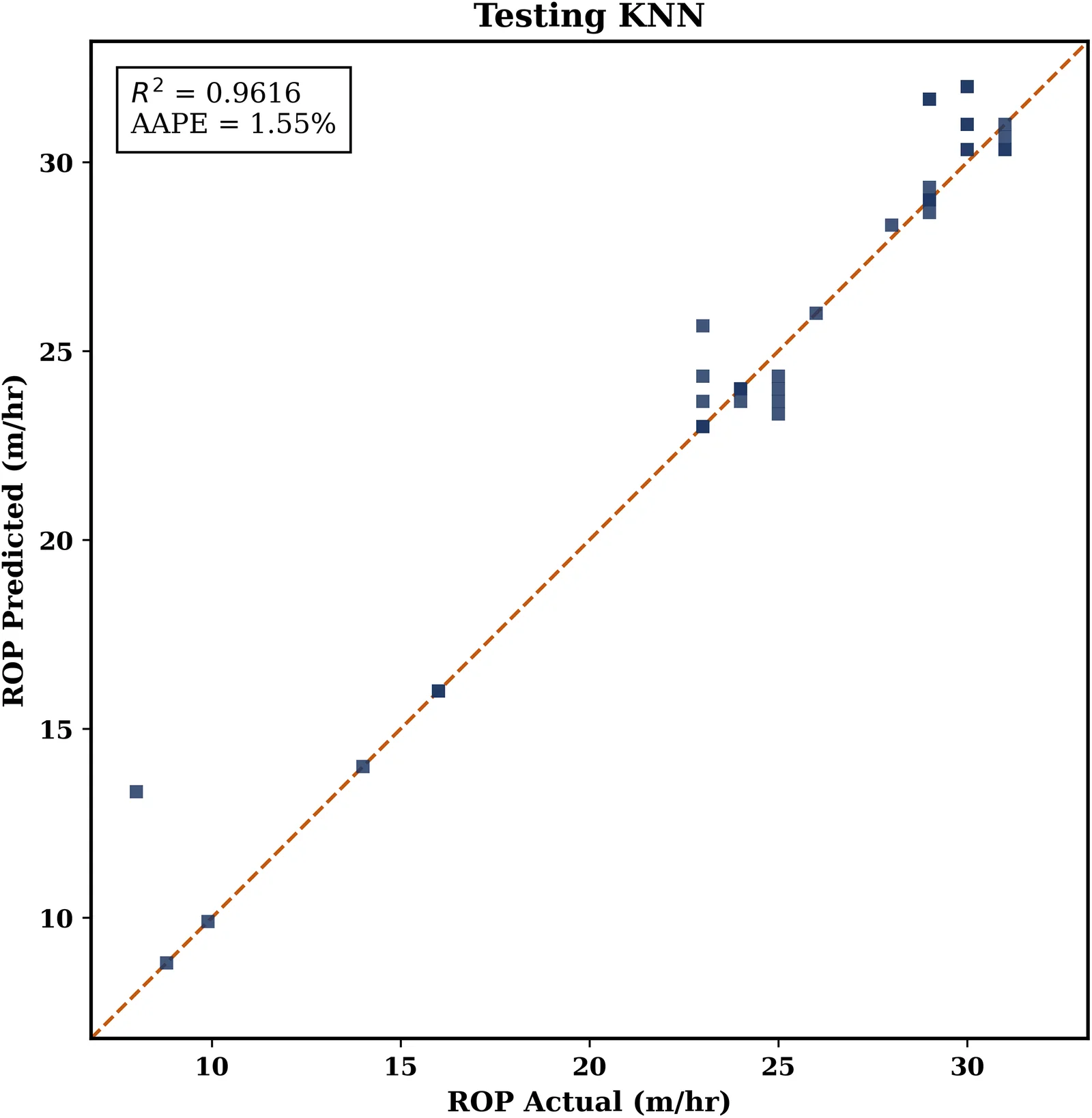
Cross-plots of KNN model predictions for the testing dataset in scenario 2. Predicted versus actual ROP for KNN on the testing dataset, Scenario 2 with R^2^ = 0.9616 and AAPE = 1.55%.

**Fig 10:**
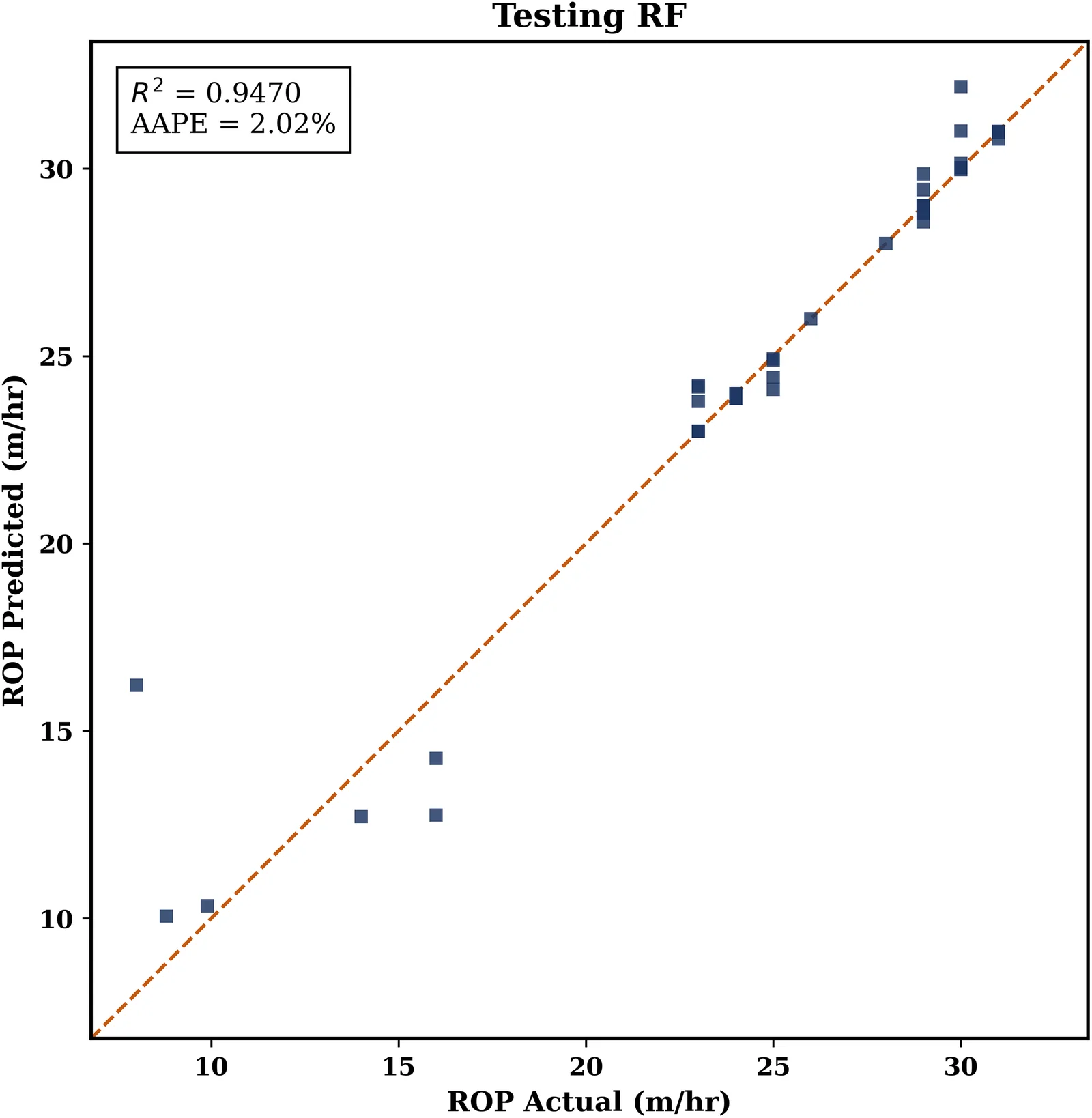
Cross-plots of RF model predictions for the testing dataset in scenario 2. Predicted versus actual ROP for RF on the testing dataset, Scenario 2 with R^2^ = 0.9470 and AAPE = 2.02%.

A slight reduction in torque decreased GBR’s and RF’s accuracy but improved KNN’s accuracy, R^2^ increased from 0.9363 to 0.9616, which is consistent with torque’s mechanical correlation with WOB and RPM. This removes redundant input, which reduces noise in the KNN’s Euclidean distance metric, sharpening its neighborhood structure, a pattern consistent with reduced-feature ROP models in the literature that obtain strong accuracy using only WOB, RPM, and flow-related parameters [20,55]. The smaller relative drop in GBR as compared to RF indicates that boosting might be more capable of reassigning importance to the other informative features when a correlated input is removed.

#### 3.1.3. Scenario 3.

Table 15 presents the Bayesian optimization results for models with both average MW and torque removed, compared to the previous optimization, which removed only torque. The performance of the GBR model remains high, with a testing R^2^ of 0.9805 and AAPE of 1.45%. This indicates that removing average MW did not significantly impact the model’s predictive accuracy, and the overall performance remains stable.

**Table 15:** Best hyperparameters in scenario 3.

Model	Parameter	Optimal Value
GBR	n_estimators	182
	learning_rate	0.2
	max_depth	7
	subsample	0.5
	min_samples_leaf	5
	min_samples_split	30
RF	n_estimators	75
	max_depth	15
	min_samples_leaf	3
	min_samples_split	5
KNN	n_neighbors	3
	leaf_size	8

Figs 11–13 present the cross plots across the three machine learning models in scenario 3. The GBR model demonstrates a high level of accuracy, with the points converging in the perfect line. The other models, KNN and RF also achieved high coefficient correlation with a few points scattering from perfect line. This indicates that removing average MW did not significantly impact the model’s predictive accuracy. The Tables 16 and 17 summarize the results for scenario 3 on training and testing datasets.

**Fig 11:**
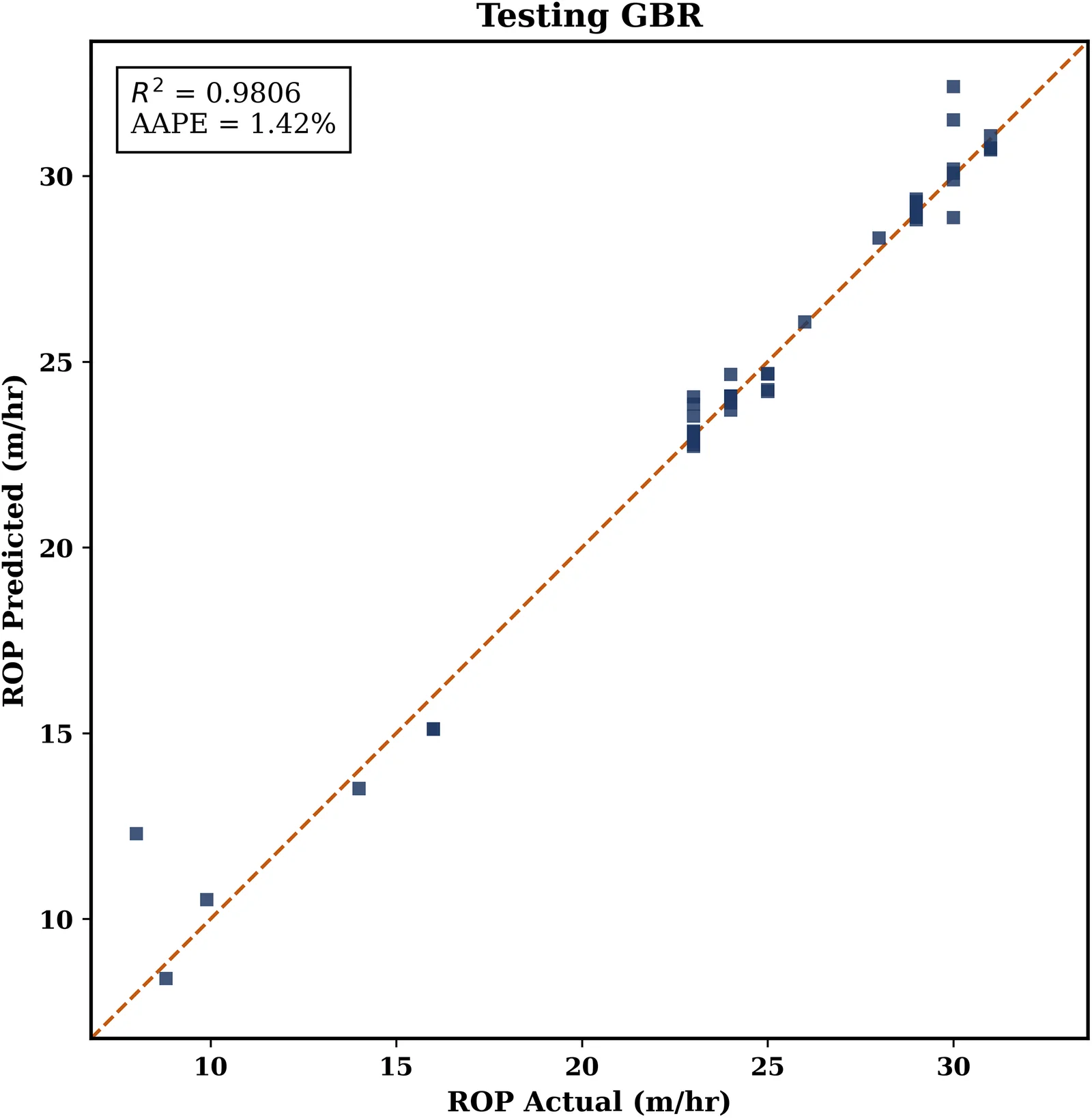
Cross-plots of GBR model predictions for the testing dataset in scenario 3. Predicted versus actual ROP for GBR on the testing dataset, Scenario 3 with R^2^ = 0.9806 and AAPE = 1.42%.

**Fig 12:**
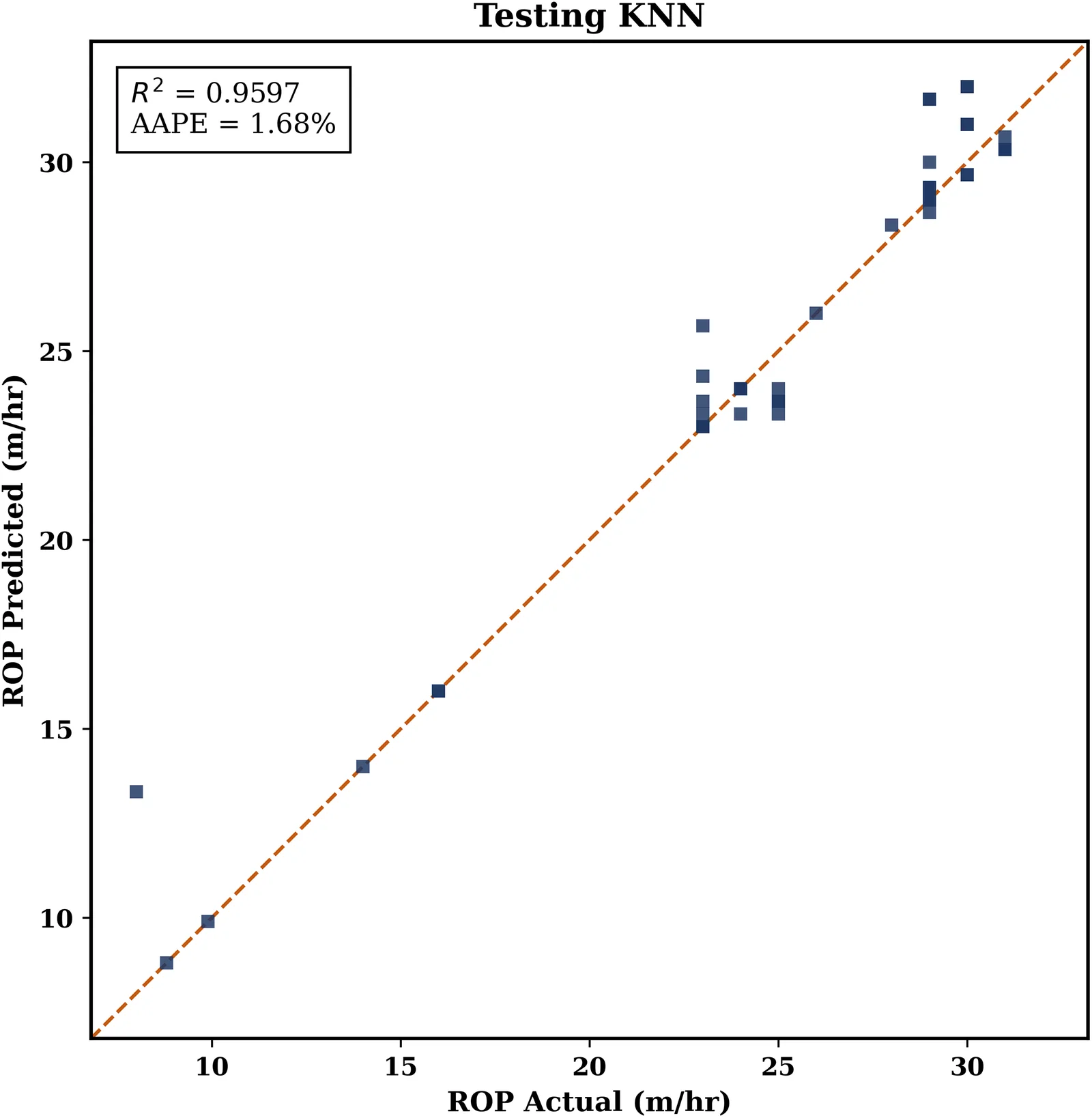
Cross-plots of KNN model predictions for the testing dataset in scenario 3. Predicted versus actual ROP for KNN on the testing dataset, Scenario 3 with R^2^ = 0.9597 and AAPE = 1.68%.

**Fig 13:**
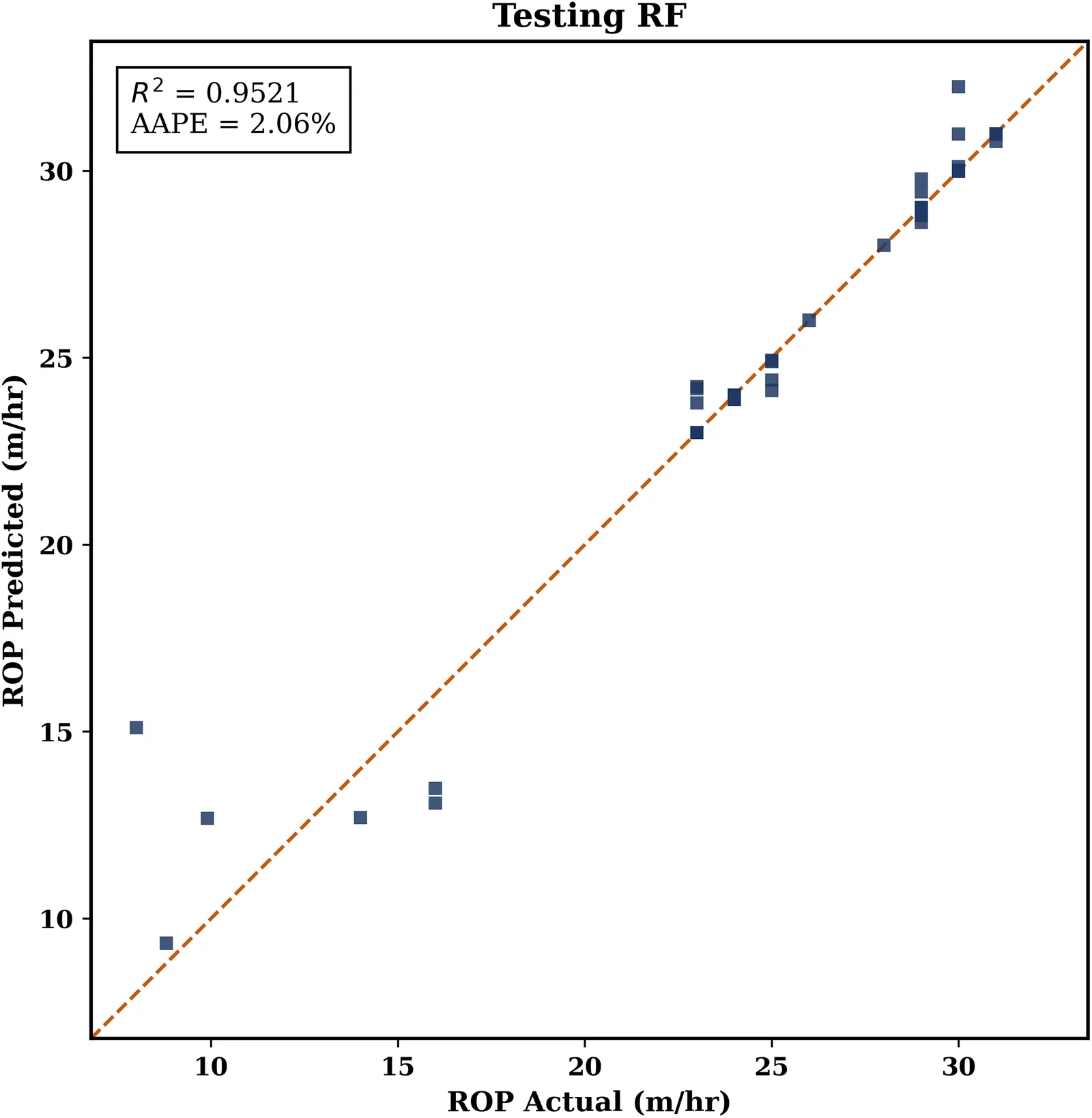
Cross-plots of RF model predictions for the testing dataset in scenario 3. Predicted versus actual ROP for RF on the testing dataset, Scenario 3 with R^2^ = 0.9521 and AAPE = 2.06%.

**Table 16:** Statistical analyses for training dataset (scenario 3).

Model	R^2^	RMSE	AAPE, %
GBR	0.9959	0.316	0.744
RF	0.9801	0.626	1.17
KNN	0.9803	0.659	1.46

**Table 17:** Statistical analyses for testing dataset (scenario 3).

Model	R^2^	RMSE	AAPE, %
GBR	0.9805	0.591	1.42
RF	0.9597	0.851	1.68
KNN	0.9521	0.927	2.06

The near identical accuracy for GBR after removing both torque and mud weight suggests that WOB, RPM, depth, and flow hold most of the predictive signal for ROP in this well. This is compatible with feature importance findings reported in the literatures, where a small subset of mechanical and formation‐related parameters, rather than the full available feature set, typically accounts for most of the explained variance [1,55]. The intermediate performance of KNN exemplifies the non-monotonic sensitivity of instance-based learners to feature selection.

### 3.2. SHAP analysis

To interpret feature contributions beyond permutation importance, SHAP (SHapley Additive exPlanations) values were computed for all three models across all three feature scenarios. SHAP measures the marginal contribution of each feature to individual predictions, capturing the direction and magnitude of influence, based on cooperative game theory [56] and adapted to machine learning [57]. For GBR and RF, SHAP values were calculated exactly using TreeExplainer [58], for KNN, values were approximated using KernelExplainer, a method appropriate for non-tree estimators. Figs 14–22 show the results for each scenario and model.

**Fig 14:**
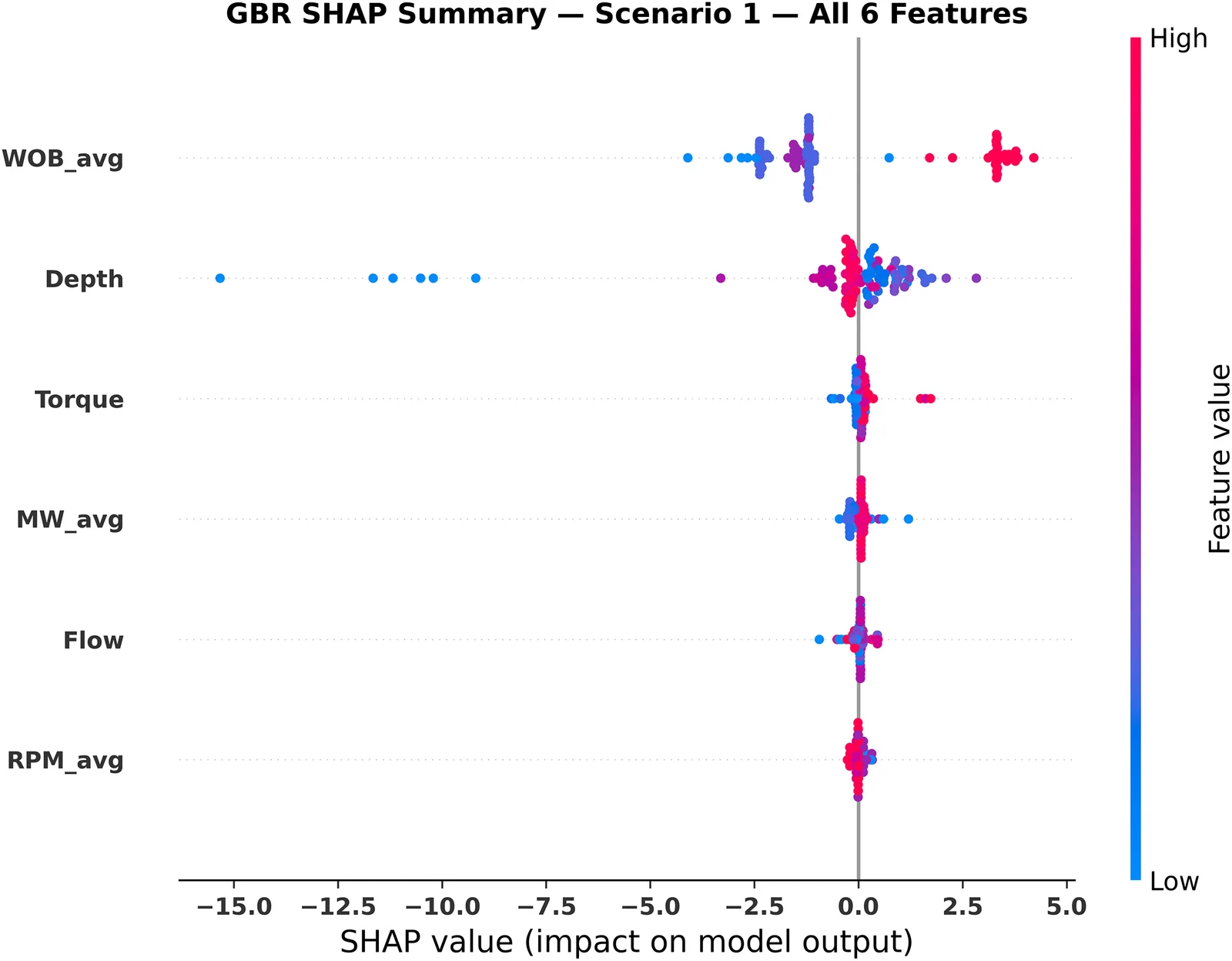
SHAP analysis of scenario 1 of GBR model. WOB is the most influential feature, and high WOB values push predicted ROP upward.

**Fig 15:**
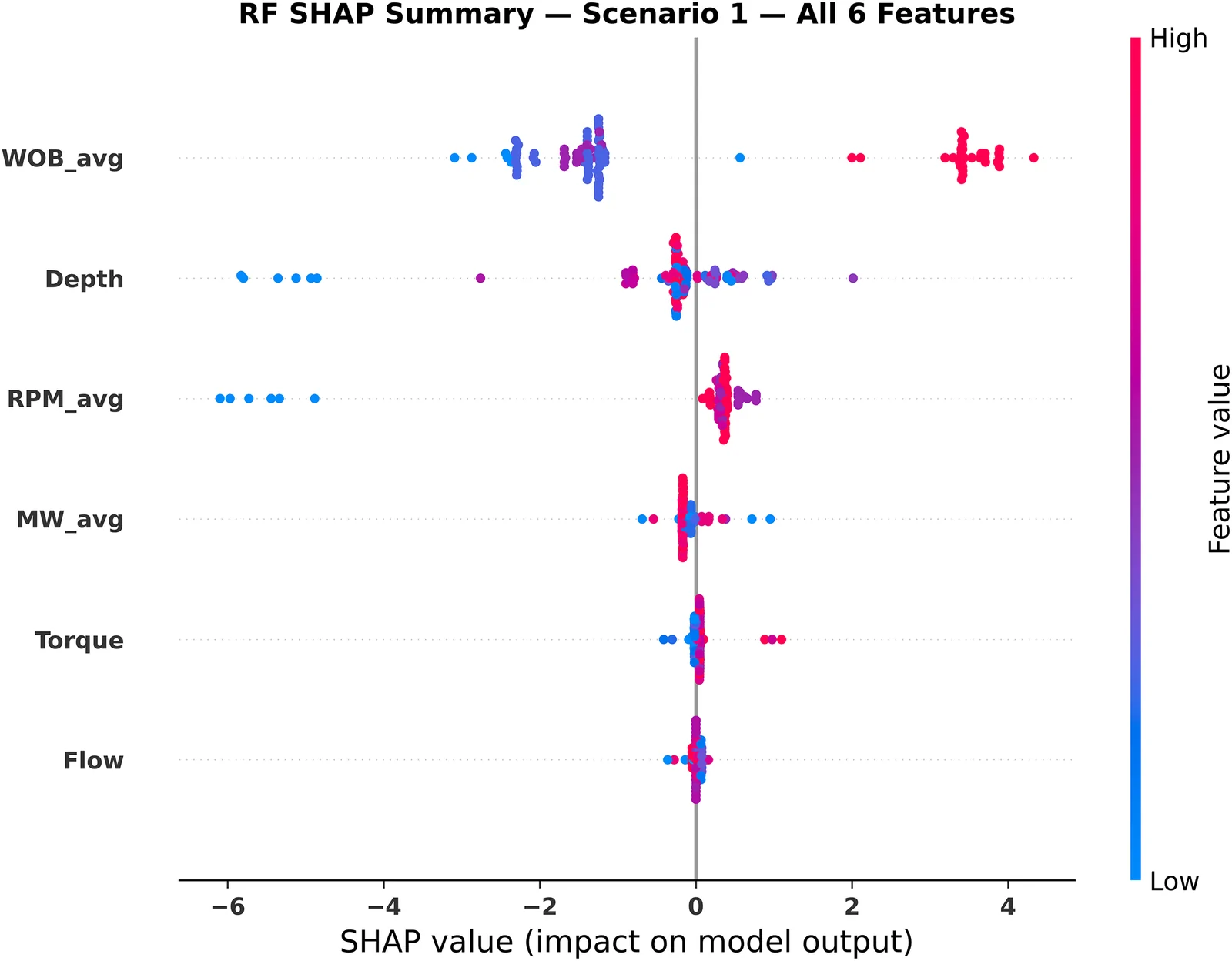
SHAP analysis of scenario 1 of RF model. WOB dominates, followed by depth and RPM, with high WOB again increasing predicted ROP.

**Fig 16:**
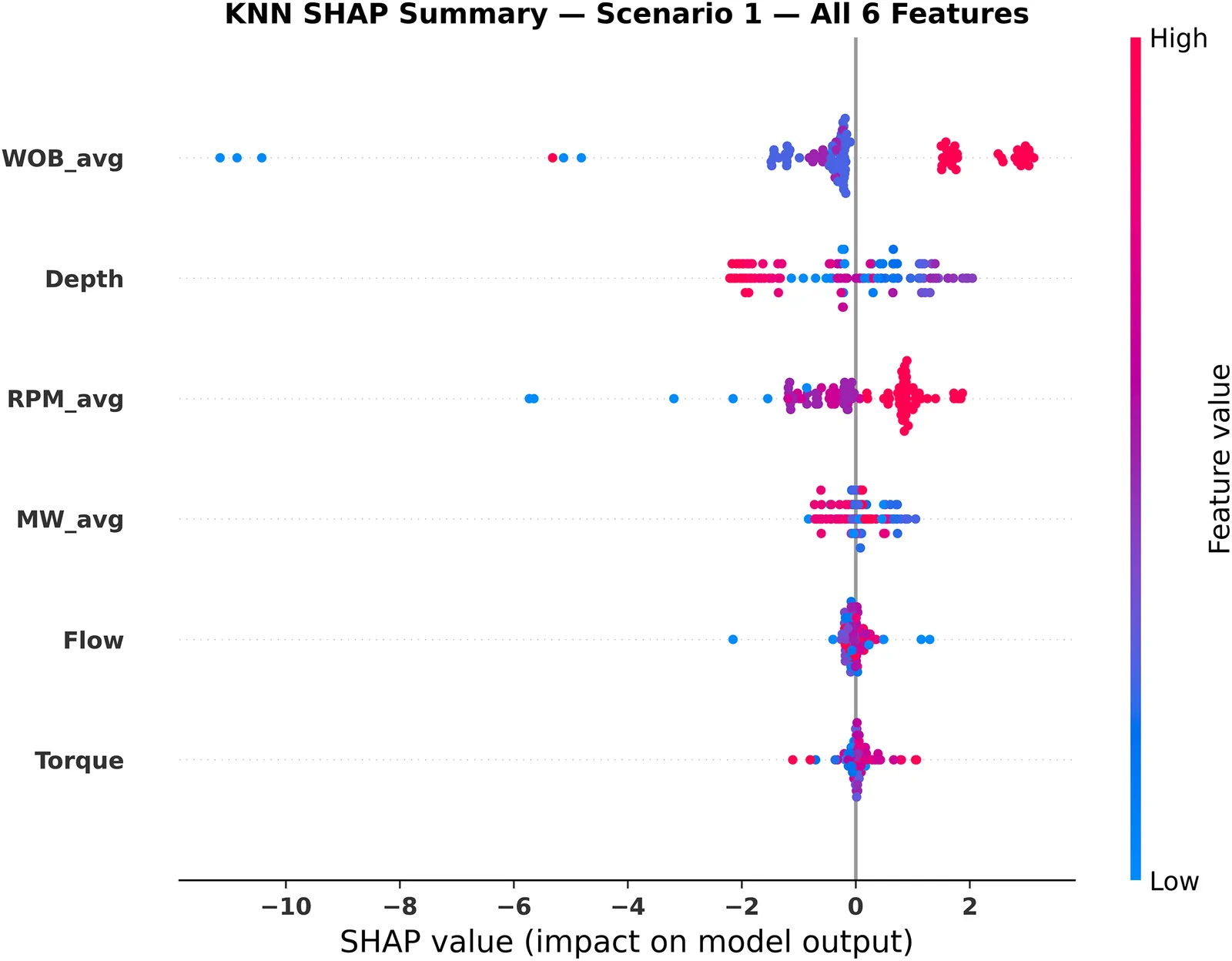
SHAP analysis of scenario 1 of KNN model. WOB is the dominant feature, followed by depth and RPM.

**Fig 17:**
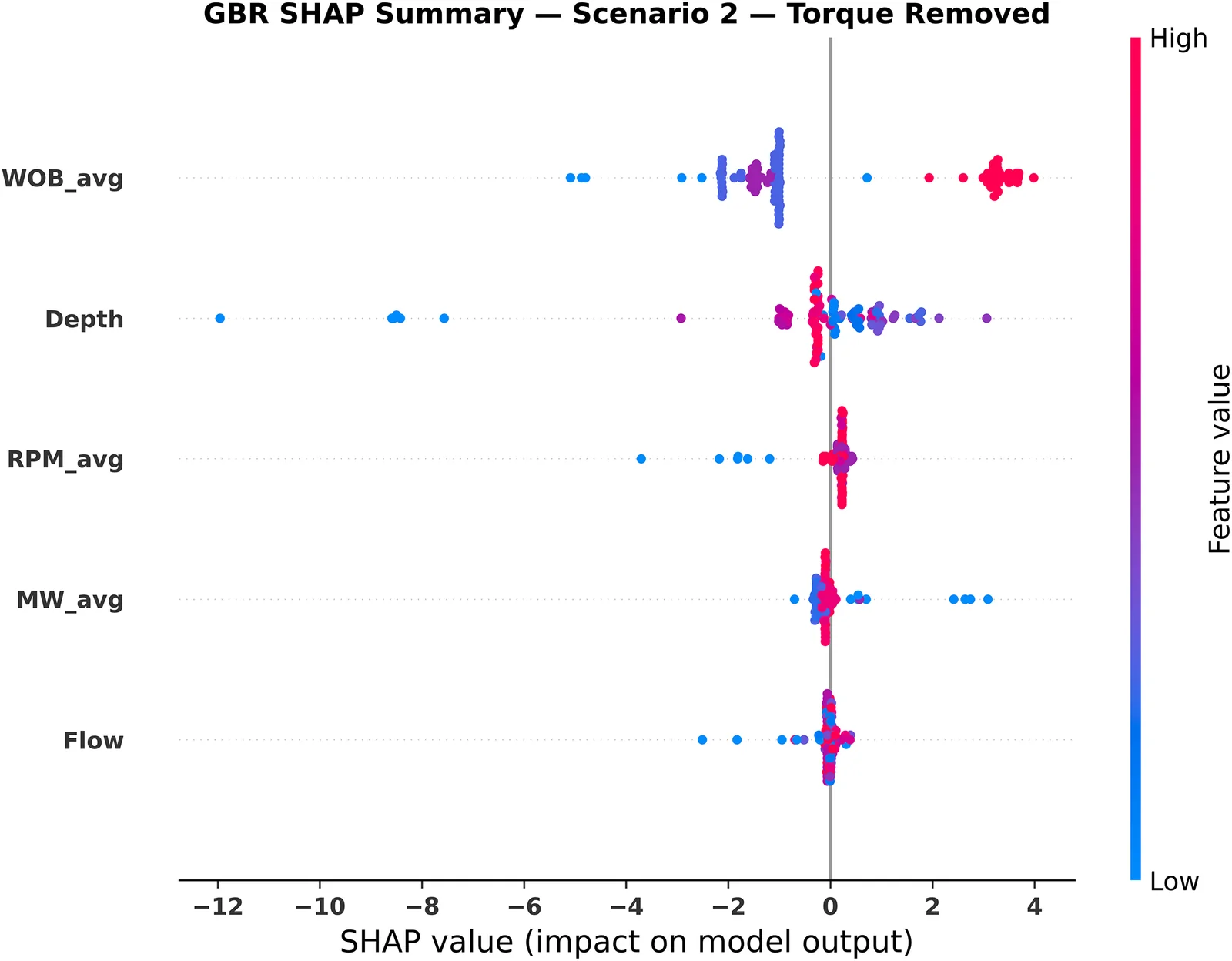
SHAP analysis of scenario 2 of GBR model. WOB and depth remain the top two features, with RPM, MW and flow rate contributing progressively less.

**Fig 18:**
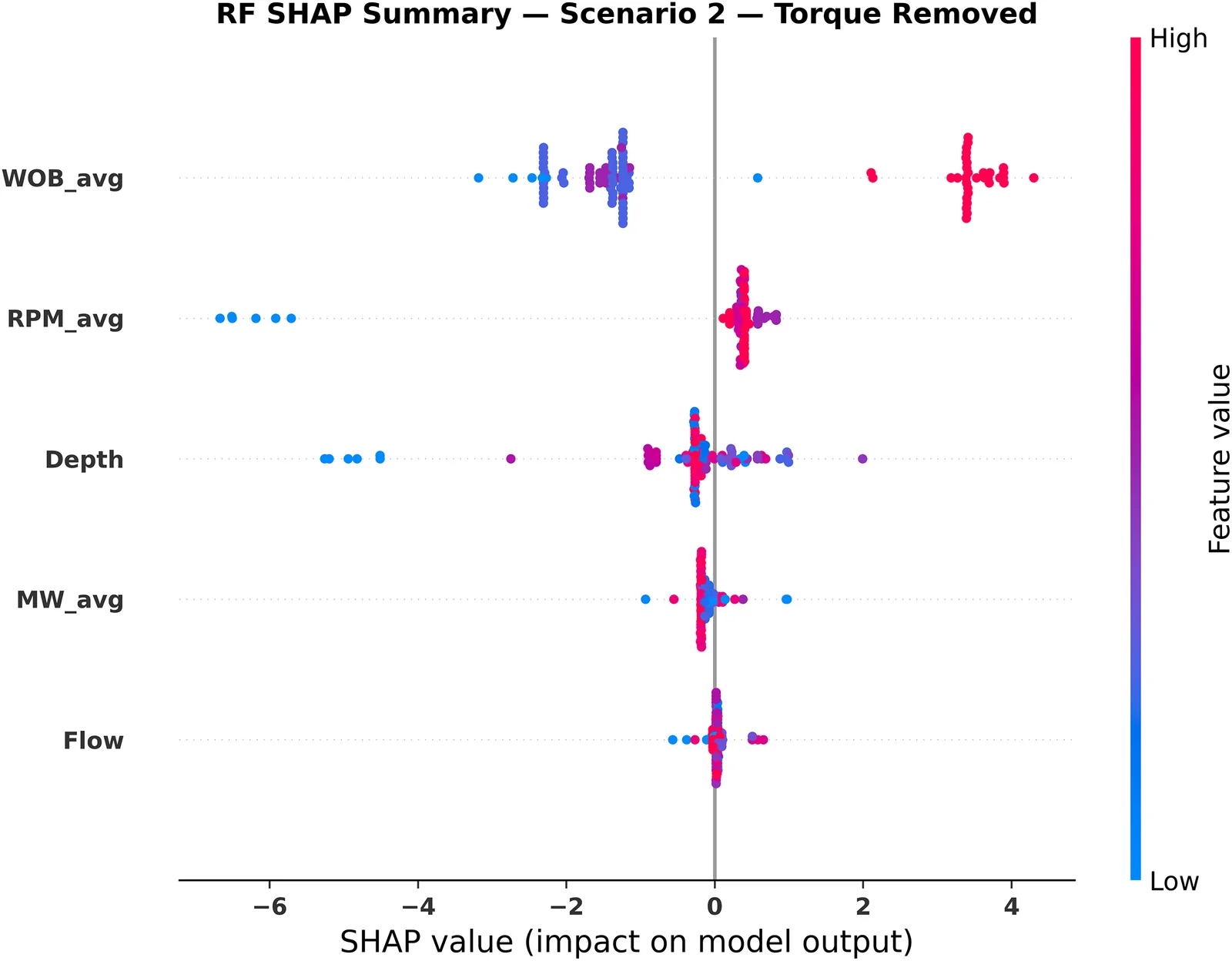
SHAP analysis of scenario 2 of RF model. WOB is the dominant feature, and RPM ranks above depth, unlike the GBR and KNN models.

**Fig 19:**
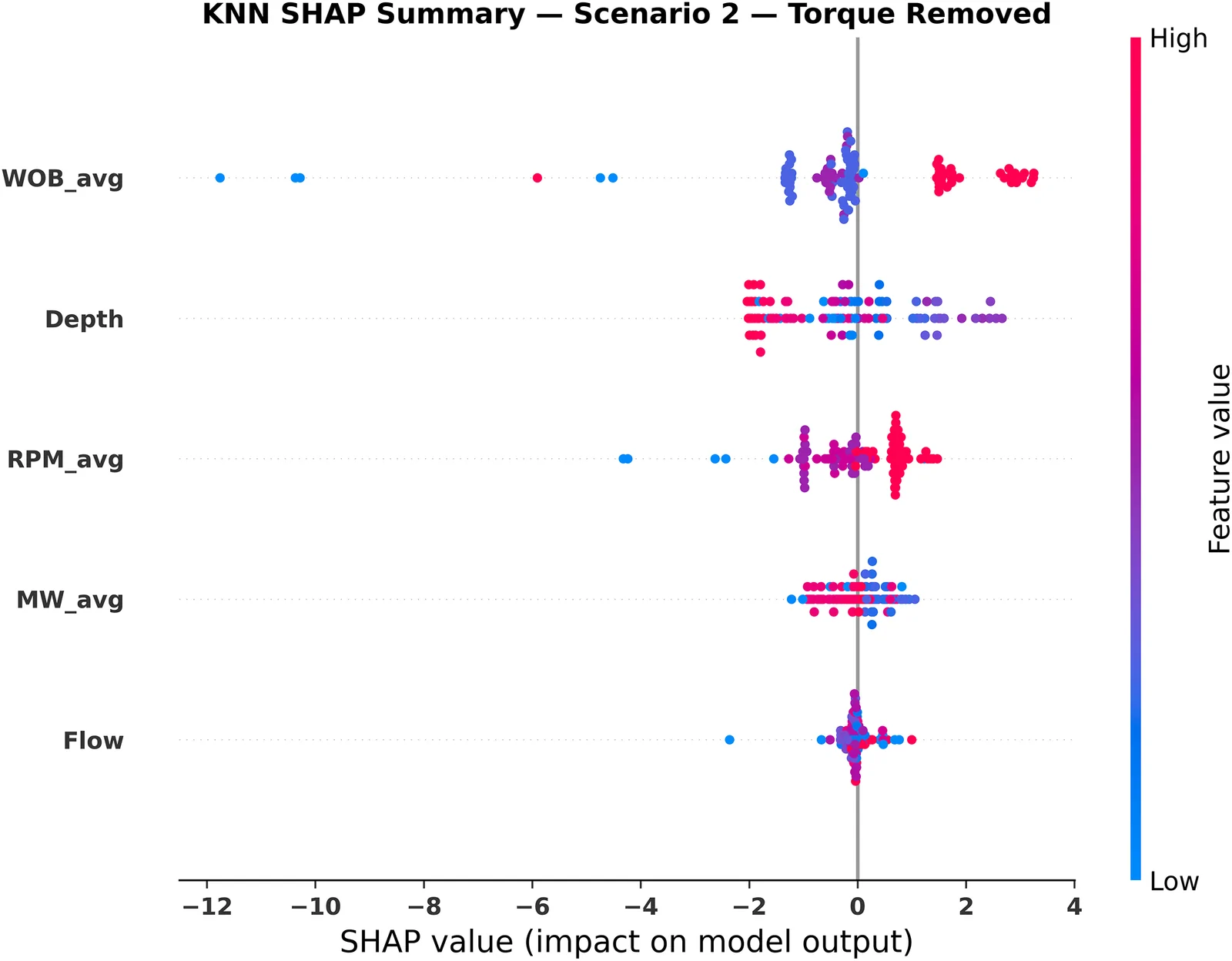
SHAP analysis of scenario 2 of KNN model. WOB is the most influential feature, followed by depth and RPM, with MW and flow rate contributing least.

**Fig 20:**
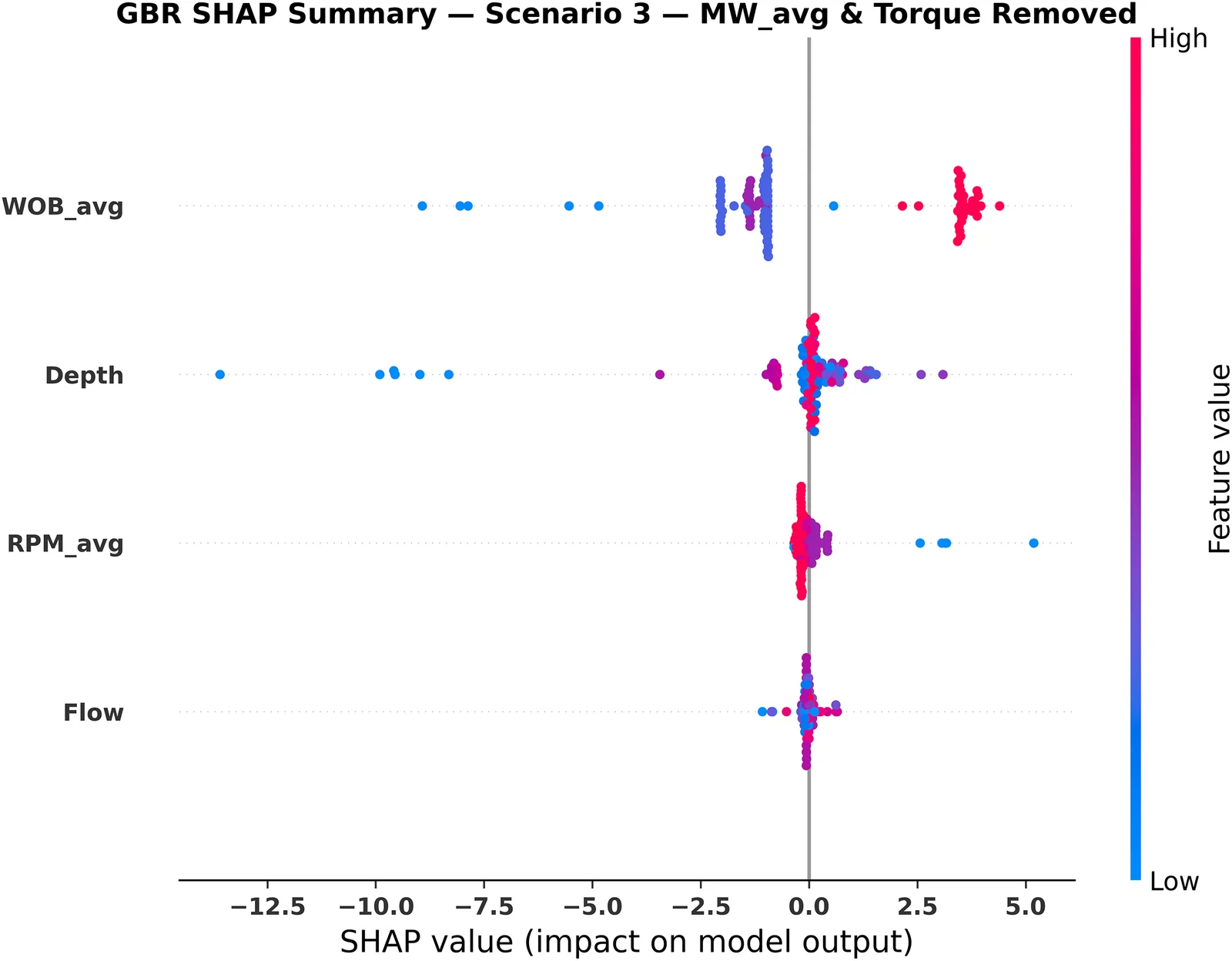
SHAP analysis of scenario 3 of GBR model. WOB is the main driver of predicted ROP, followed by depth, RPM and flow rate.

**Fig 21:**
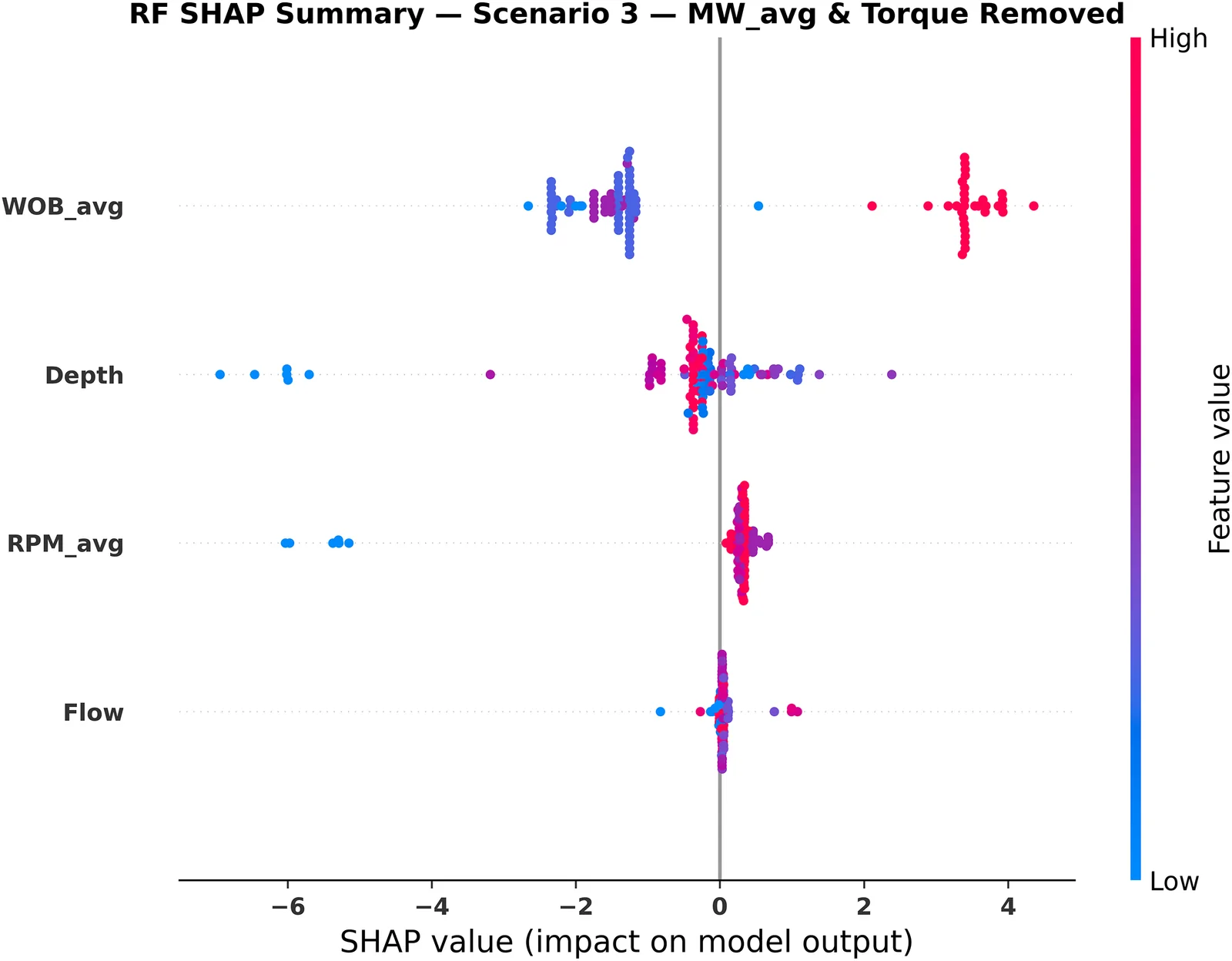
SHAP analysis of scenario 3 of RF model. WOB dominates, followed by depth and RPM, with flow rate having the smallest effect.

**Fig 22:**
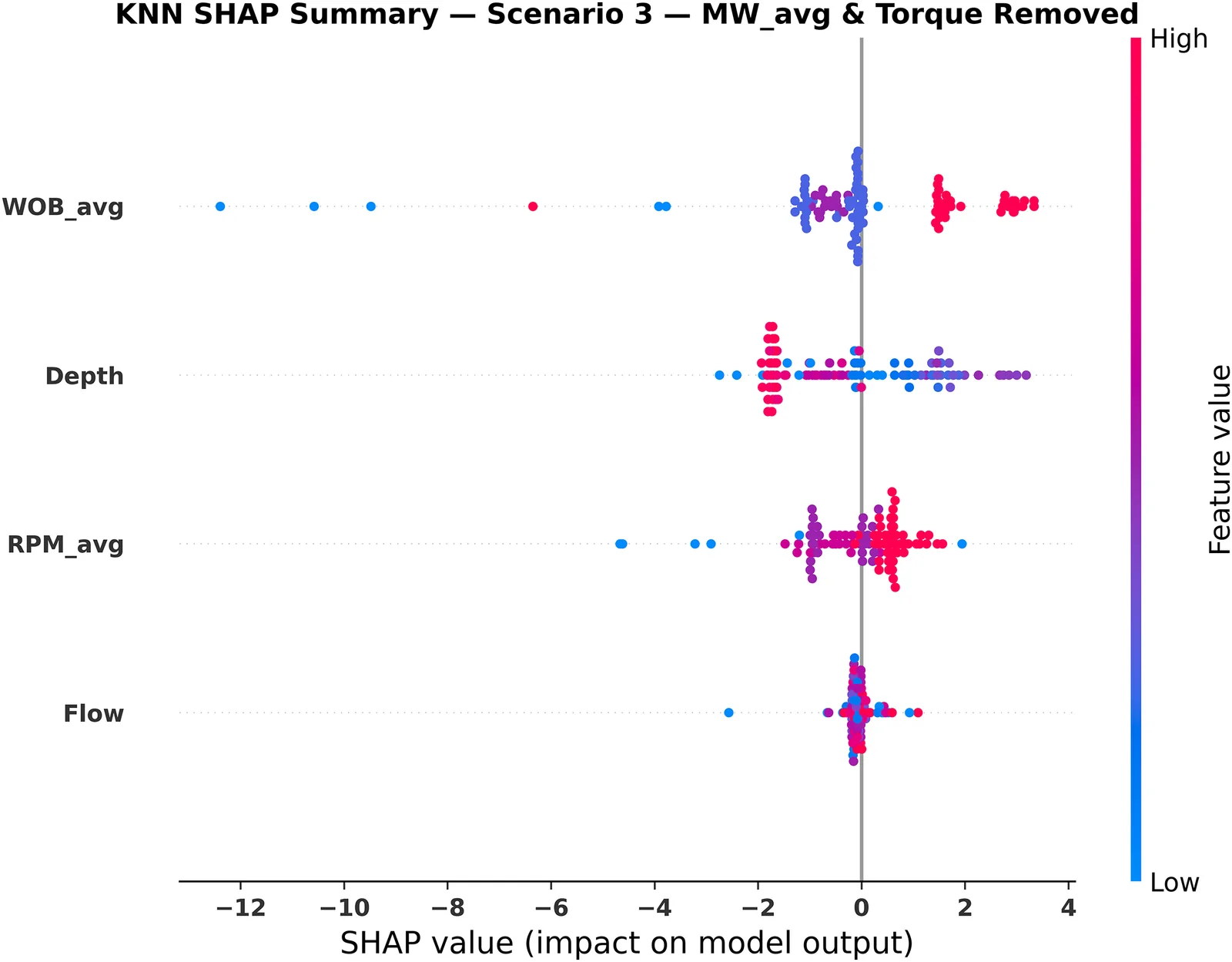
SHAP analysis of scenario 3 of KNN model. The feature ranking is WOB, depth, RPM, then flow rate, consistent with the other models.

In all three scenarios, the two most important predictors of ROP were consistently WOB_avg_ and depth. WOB_avg_ showed a clear monotonic trend, with higher WOB_avg_ values leading to higher predicted ROP. The effect of depth was more scattered, consistent with depth being an indirect proxy for lithology rather than a direct mechanical driver. This ranking was still maintained when removing torque (Scenario 2) and MW_avg_ (Scenario 3), indicating that the interpretability results are robust to feature subset. Importantly, RPM_avg_ was higher for KNN than GBR or RF in all scenarios, most likely because KNN is more sensitive to the distance in feature space rather than a more significant physical effect, consistent with the permutation importance results.

### 3.3. Trend analysis

The purpose of trend analysis is to assess whether the models’ outputs are scientifically sound [59]. In our models, we investigate the effects of varying WOB and rotary speed (N) while keeping other parameters constant. Although a model may replicate parameter relationships during training and yield accurate predictions during testing, it may still fail to predict in a manner consistent with previously established models that are widely accepted in the industry. Each model in each scenario exhibits a different trend pattern because the number of outputs was reduced. The effects of Weight on Bit (WOB) and rotary speed on rate of penetration (ROP), proposed by Bourgoyne et al. [60] in Applied Drilling, are illustrated in Figs 23 and 24.

**Fig 23:**
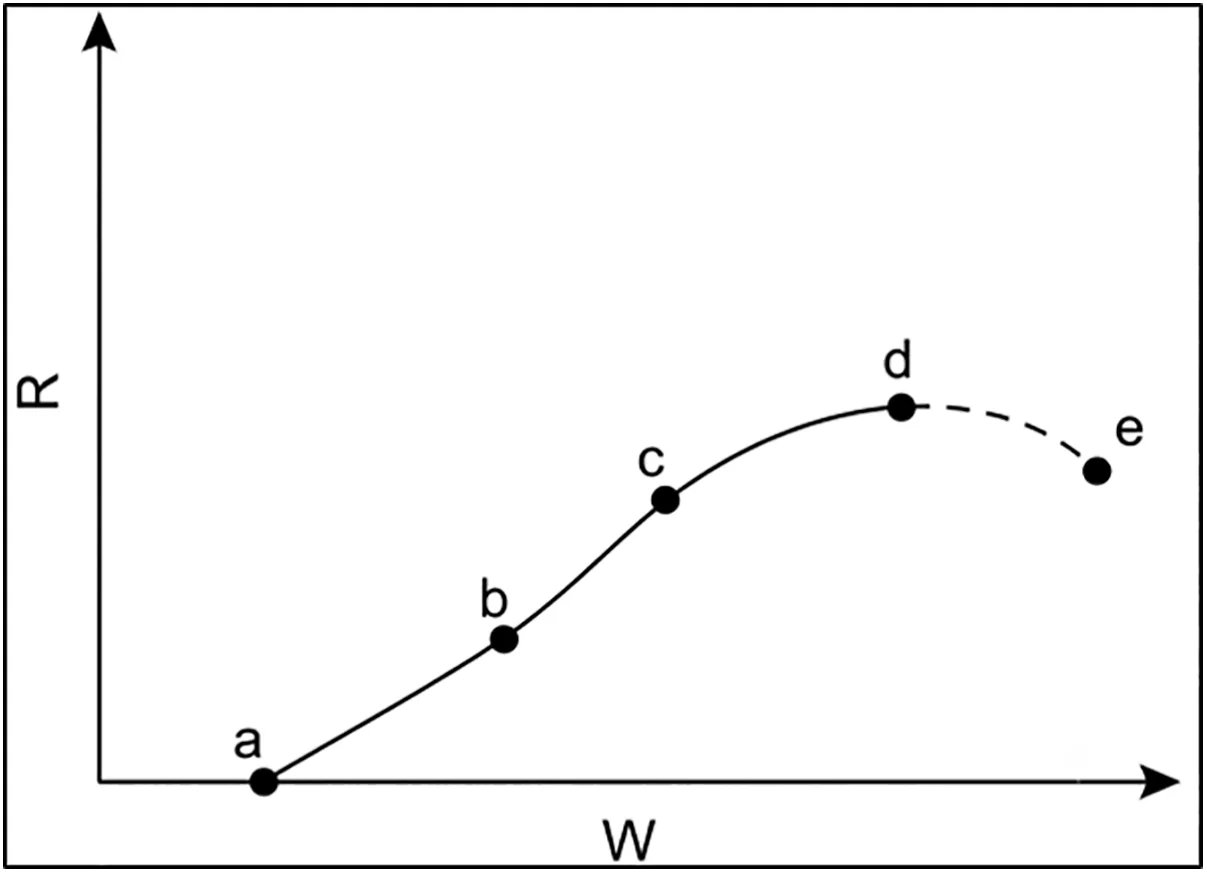
Effect of weight on bit on rate of penetration proposed by Bourgoyne et al. in applied drilling engineering [61]. ROP starts at a threshold WOB (a), increases with WOB through points b and c, and reaches a maximum at d. Beyond d (dashed line to e), ROP declines with further increases in WOB.

**Fig 24:**
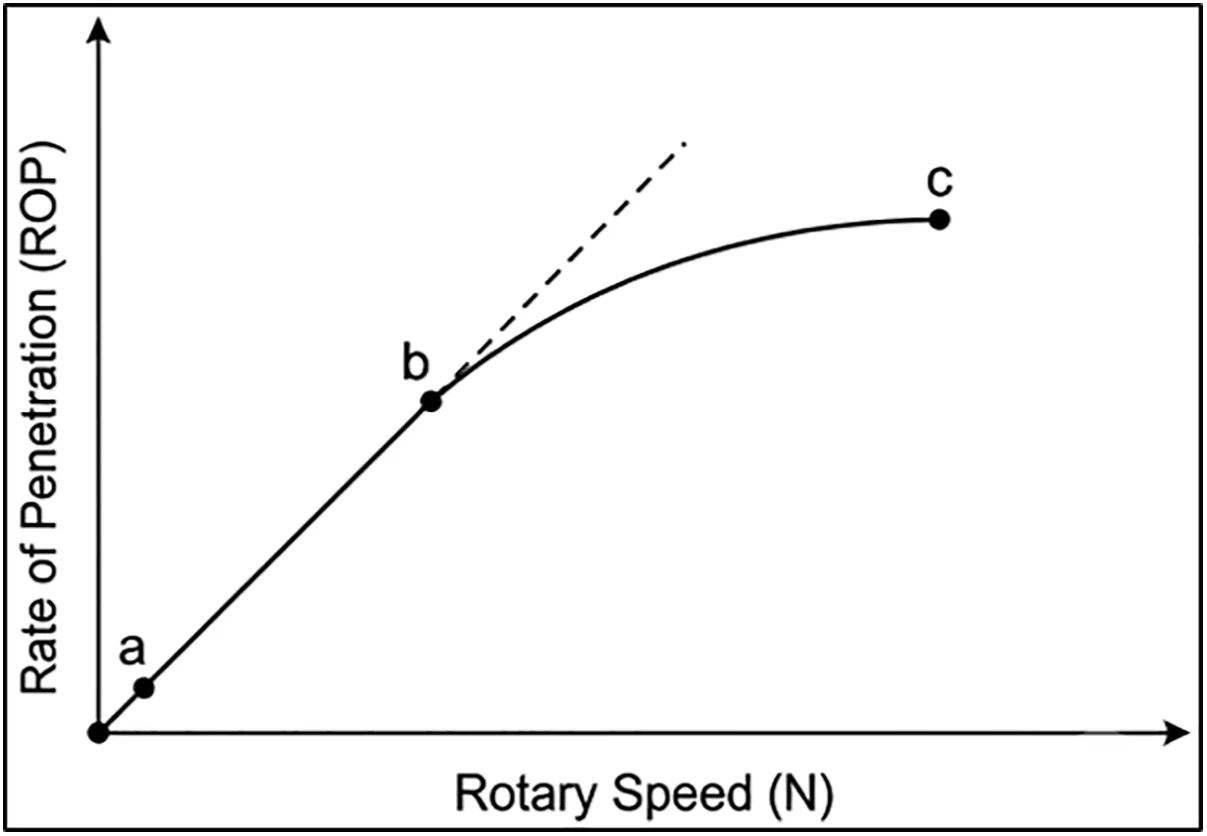
Effect of rotary speed on rate of penetration proposed by Bourgoyne et al. in applied drilling engineering [61]. ROP increases linearly with rotary speed from the origin through points a and b, then departs from the linear trend (dashed line) and levels off towards point c.

In Fig 23 at very low values of WOB, the drill bit merely grinds the rock surface, causing elastic deformation but little influence on rock breakage. Once the weight on the bit surpasses the rock’s comprehensive strength, the bit effectively scrapes and chips the rock. In this region, ROP increases linearly with increases in WOB. A maximum effective WOB is eventually reached. If too much weight is exerted, the bit matrix will not be able to penetrate any deeper formations as it experiences bit balling or floundering (point D), and poor bottom-hole cleaning will worsen this effect, causing ROP to level off or even drop. The relationship between rotary speed and ROP is shown in Fig 24. At lower rotary speed, ROP increases almost linearly with rotary speed (N). As rotational speed increases, the curve begins to deviate from the ideal linear trend. This happens because the hydraulic system struggles to clear the rapidly formed cuttings immediately. At flounder point (high rotary speed), the curve flattens out entirely at point C. Beyond this speed, ROP will stay constant regardless of the increasing rotary speed. The trend analysis of each scenario was shown Figs 25–30, respectively.

**Fig 25:**
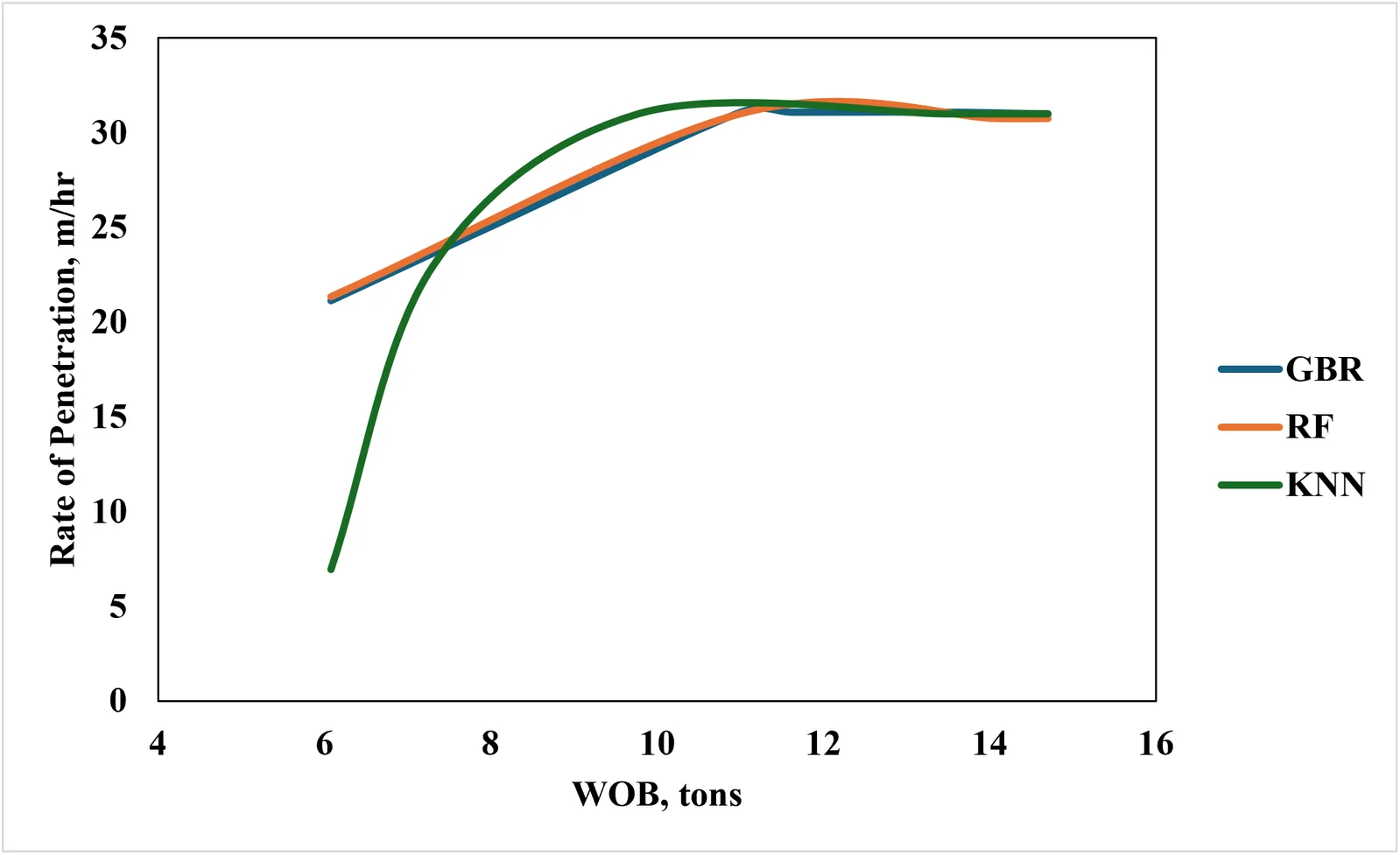
Trend analysis of effect weight on bit in scenario 1 across all the models. All three models show ROP rising with WOB and levelling off at about 31 m/hr beyond approximately 11-12 tons.

**Fig 26:**
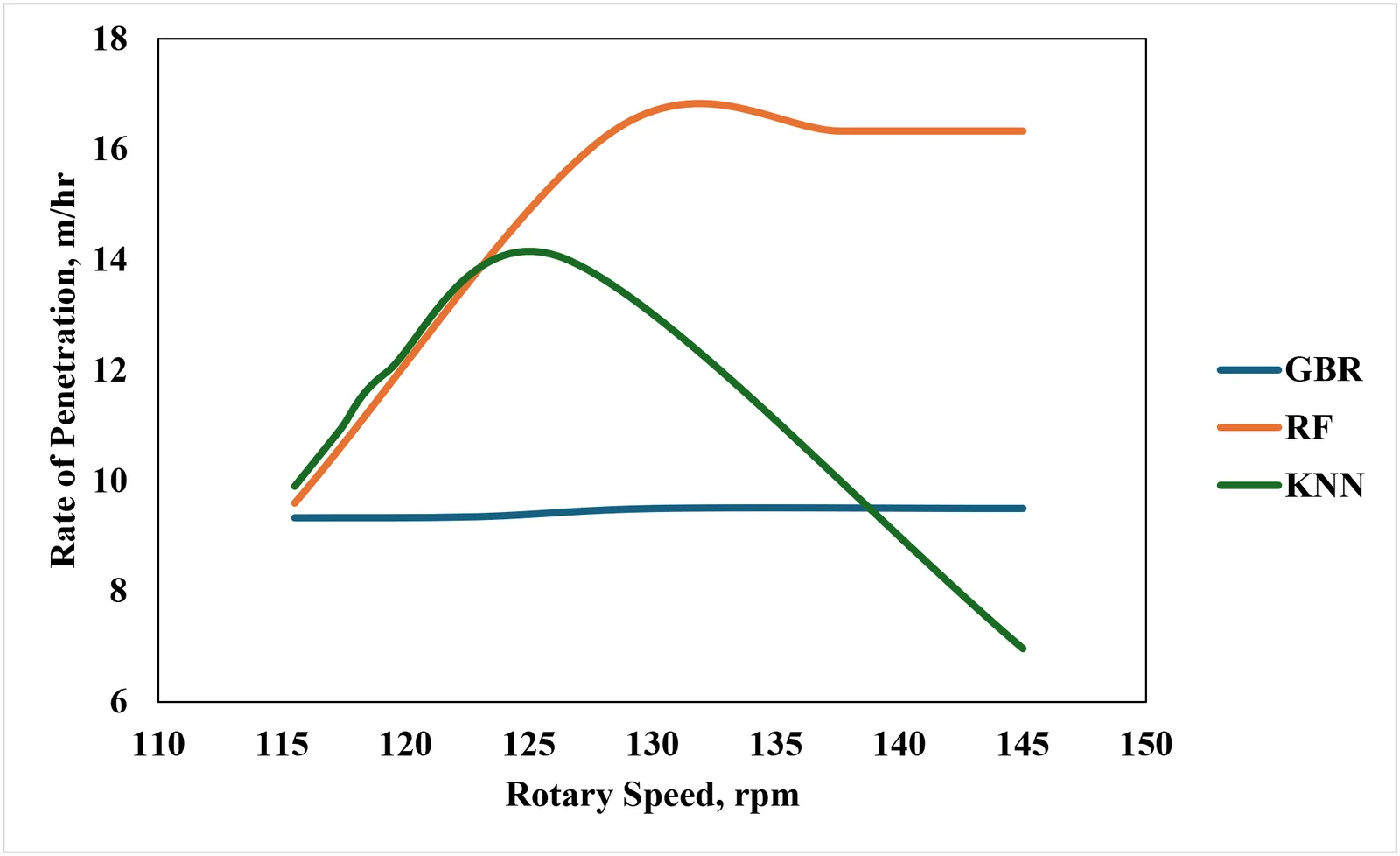
Trend analysis of effect of rotary speed in scenario 1 across all the models. The models disagree: RF rises to a plateau at about 16-17 m/hr, KNN peaks near 125 rpm and then declines, and GBR stays nearly flat at about 9.5 m/hr.

**Fig 27:**
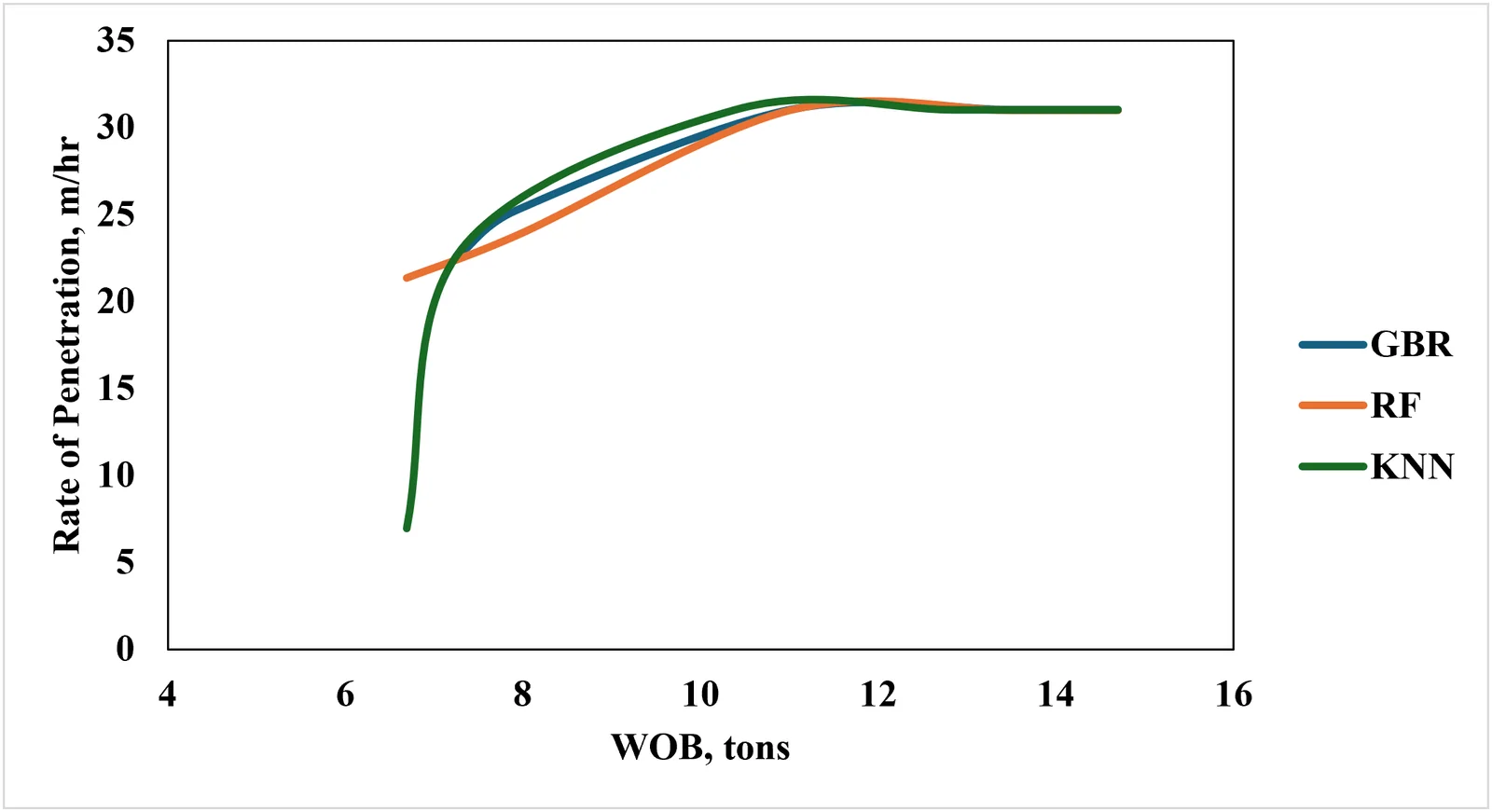
Trend analysis of effect weight on bit in scenario 2 across all the models. ROP increases with WOB and plateaus at about 31 m/hr beyond roughly 11-12 tons for all three models while KNN drops sharply at the low-WOB end.

**Fig 28:**
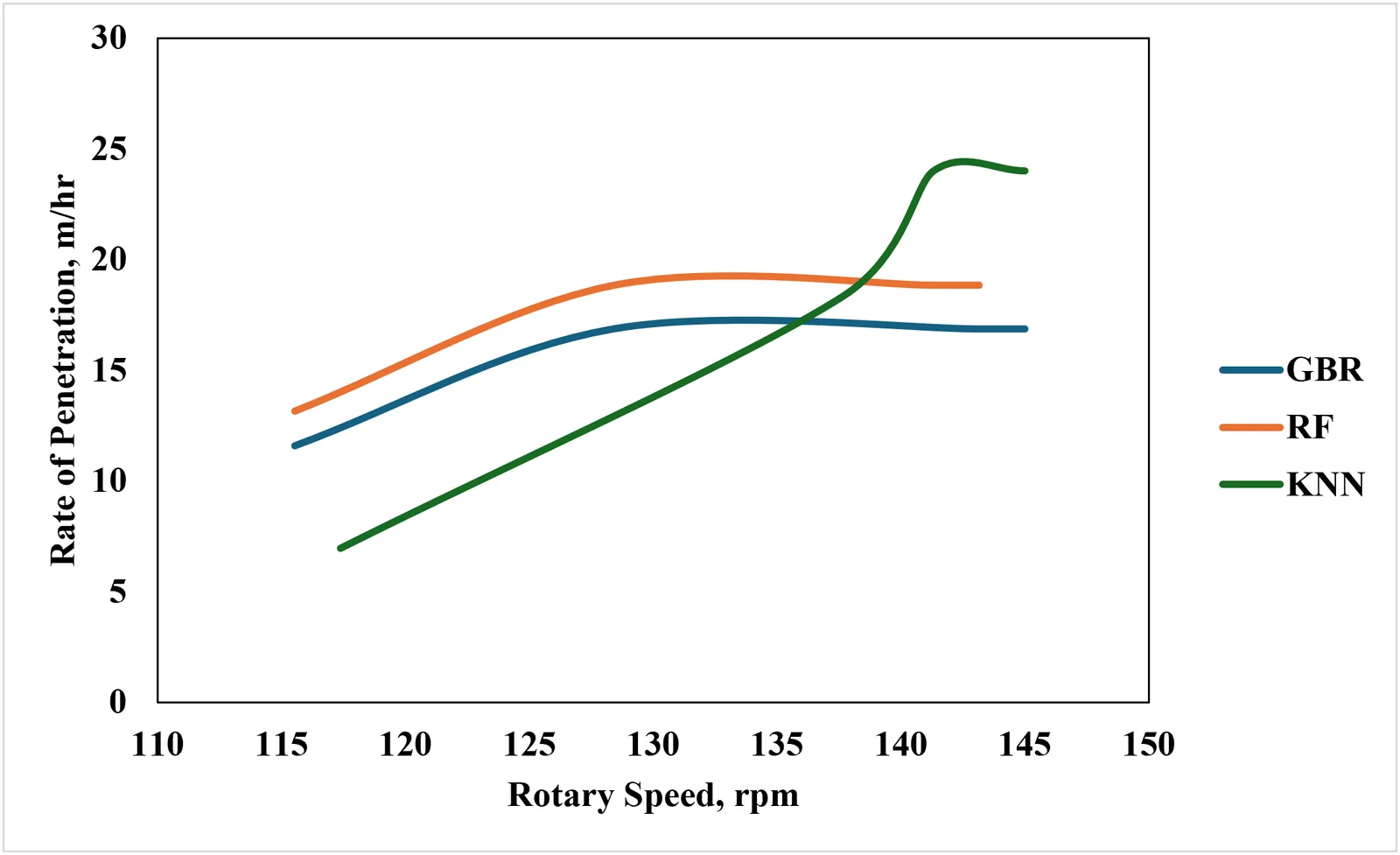
Trend analysis of effect of rotary speed in scenario 2 across all the models. GBR and RF show a gradual increase followed by a plateau near 17-19 m/hr above about 130 rpm while KNN increases almost linearly and jumps to about 24 m/hr at the highest rotary speeds.

**Fig 29:**
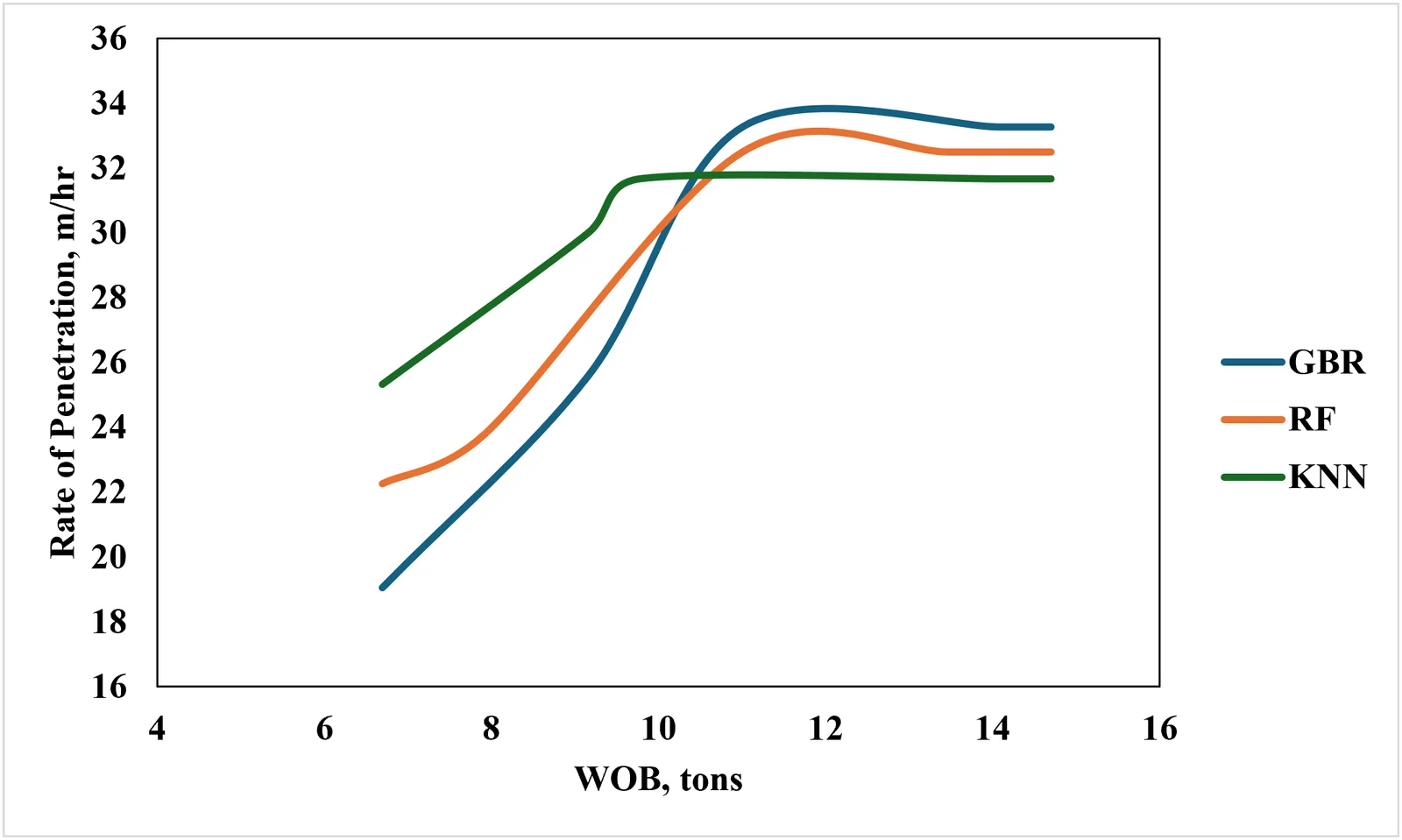
Trend analysis of effect weight on bit in scenario 3 across all the models. All models show ROP rising with WOB and plateauing at about 32-33 m/hr beyond approximately 10-11 tons, KNN predicts the highest ROP at low WOB, and GBR predicts the highest plateau.

**Fig 30:**
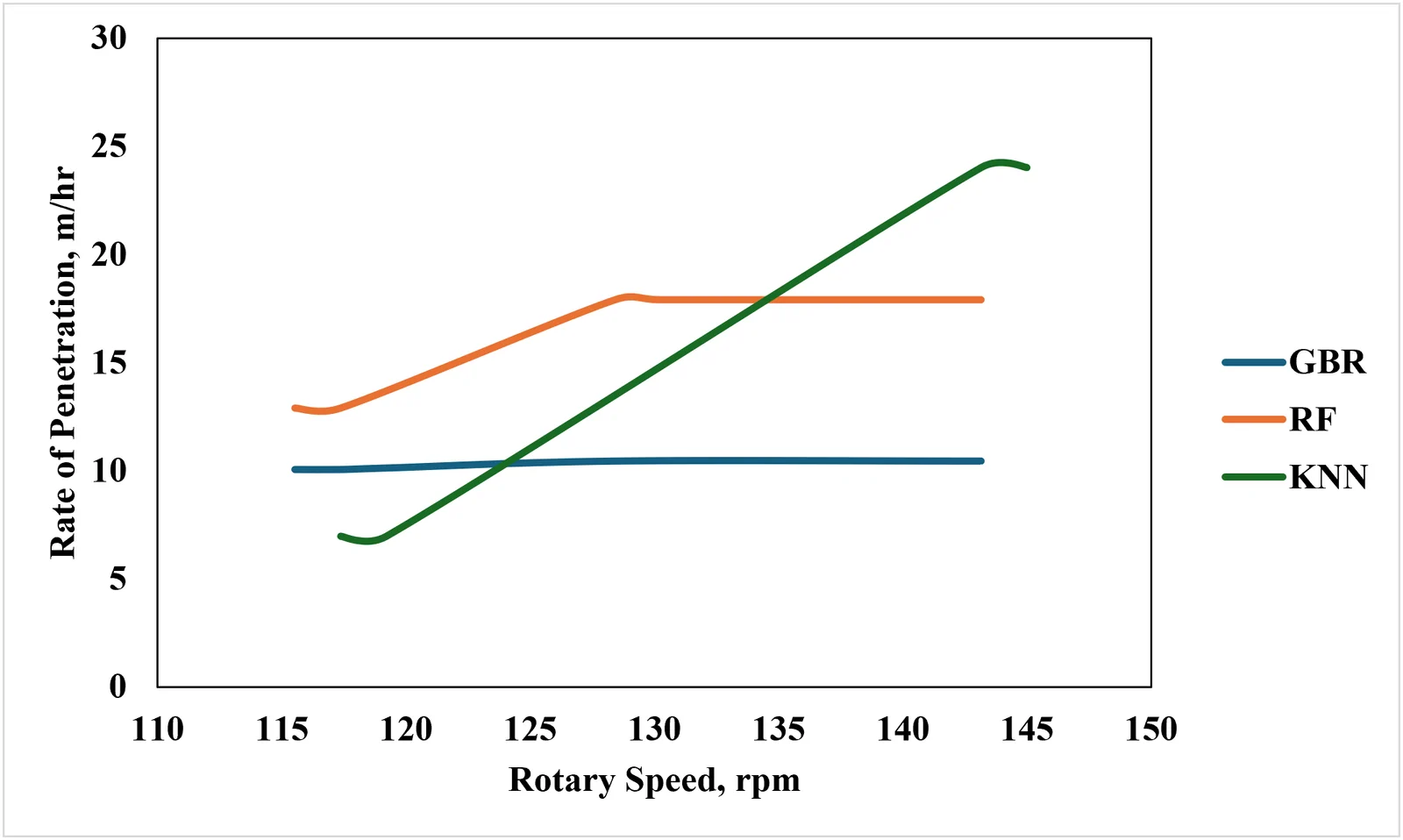
Trend analysis of effect rotary speed in scenario 3 across all the models. RF rises to about 18 m/hr and then plateaus, GBR is nearly flat at about 10 m/hr, and KNN increases almost linearly to about 24 m/hr.

The trend analysis conducted across three scenarios showed two consistent and physically meaningful patterns. First, WOB emerges as the most robustly captured variable across all models and scenarios. Every model reproduces the expected nonlinear positive relationship between WOB and ROP, with a steep increase in the low-to-mid WOB range followed by a clear plateau as consistent with Fig 23. GBR and RF tend to follow a similar pattern, exhibiting a nearly linear trend until the flounder point, except in scenario 3. However, the KNN model, particularly in scenario 1 and 2, started with a lower ROP value in similar WOB across the models.

Rotary speed (RPM) proves to be a persistent source of inter-model divergence across all scenarios. No single model produces a universally consistent RPM vs ROP trend, and the nature of the disagreement shifts across scenarios, from GBR’s complete insensitivity in scenarios 1 and 3, in which the model showed a flat/plateau line, to KNN’s monotonically unbounded increase in scenarios 2 and 3, and RF’s more physically plausible unimodal response in scenarios 1 and 2.

Trend analysis was also conducted on two well-established empirical models commonly used in industry, the Bingham model and the Bourgoyne and Young model, to see if these models can capture the expected physical drilling trends. The results of the trend analysis for the Bingham and Bourgoyne and Young models are shown in Figs 31 and 32.

**Fig 31:**
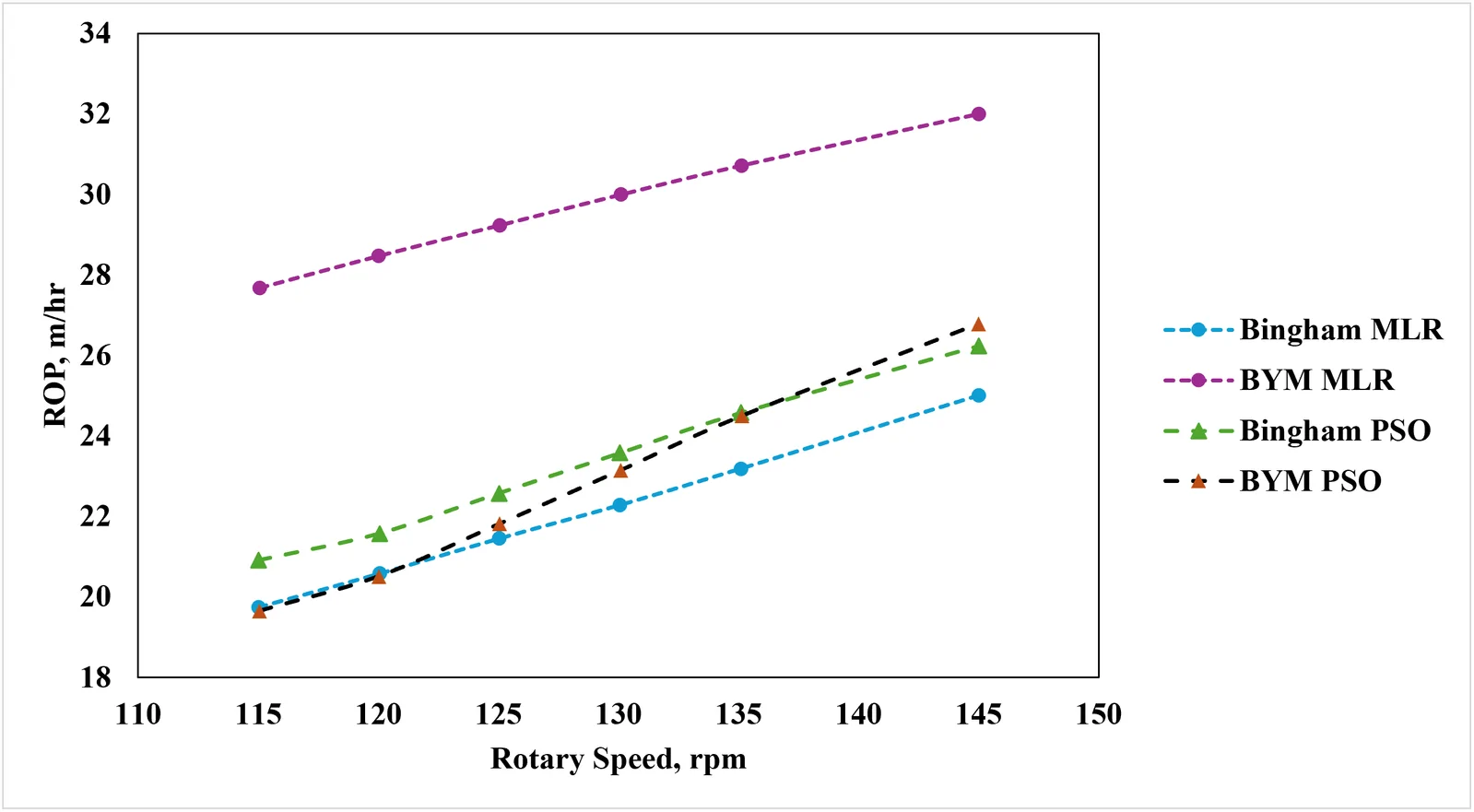
Trend analysis of effect of rotary speed for empirical models. All four curves increase almost linearly with rotary speed. BYM-MLR gives the highest ROP (about 28-32 m/hr), while the other three fall in a lower band of about 20-27 m/hr.

**Fig 32:**
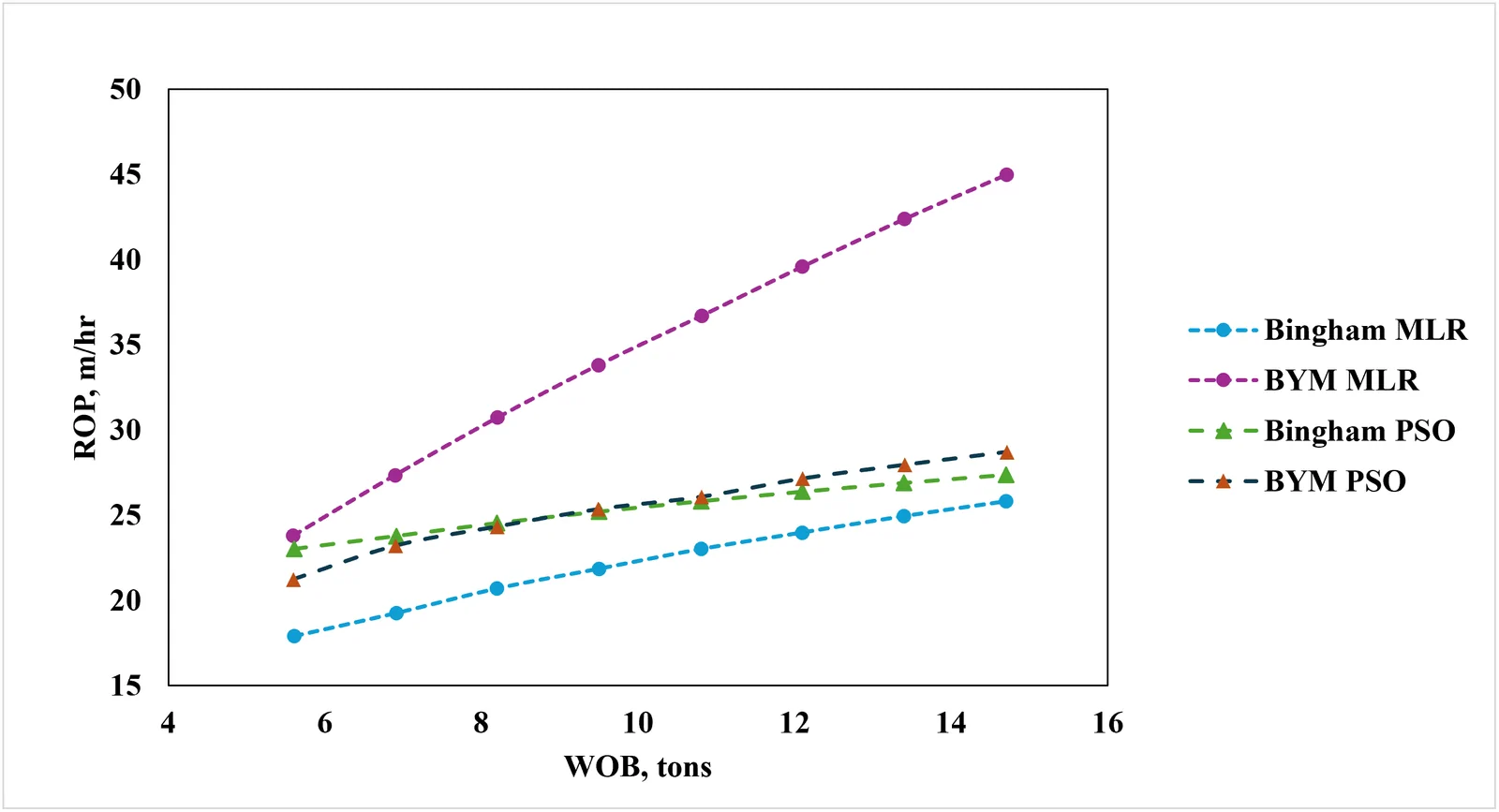
Trend analysis of effect of weight on bit for empirical models. BYM-MLR rises steeply to about 45 m/hr, while the other three models increase more gradually to about 26-29 m/hr.

From Figs 31 and 32, we can infer that neither the Bingham model nor the Bourgoyne and Young model follows the correct trend for any optimization technique. Both models give a linear correlation and thus the floundering point is not captured.

### 3.4. Empirical calculation results

The empirical models used in this work were validated using the field data. Figs 33 and 34 are cross-plots of measured rate of penetration (ROP) values from field data and calculated values using the Bingham model and Bourgoyne and Young model with two different techniques. These plots provide a visual assessment of the predictive performance of the empirical correlations.

**Fig 33:**
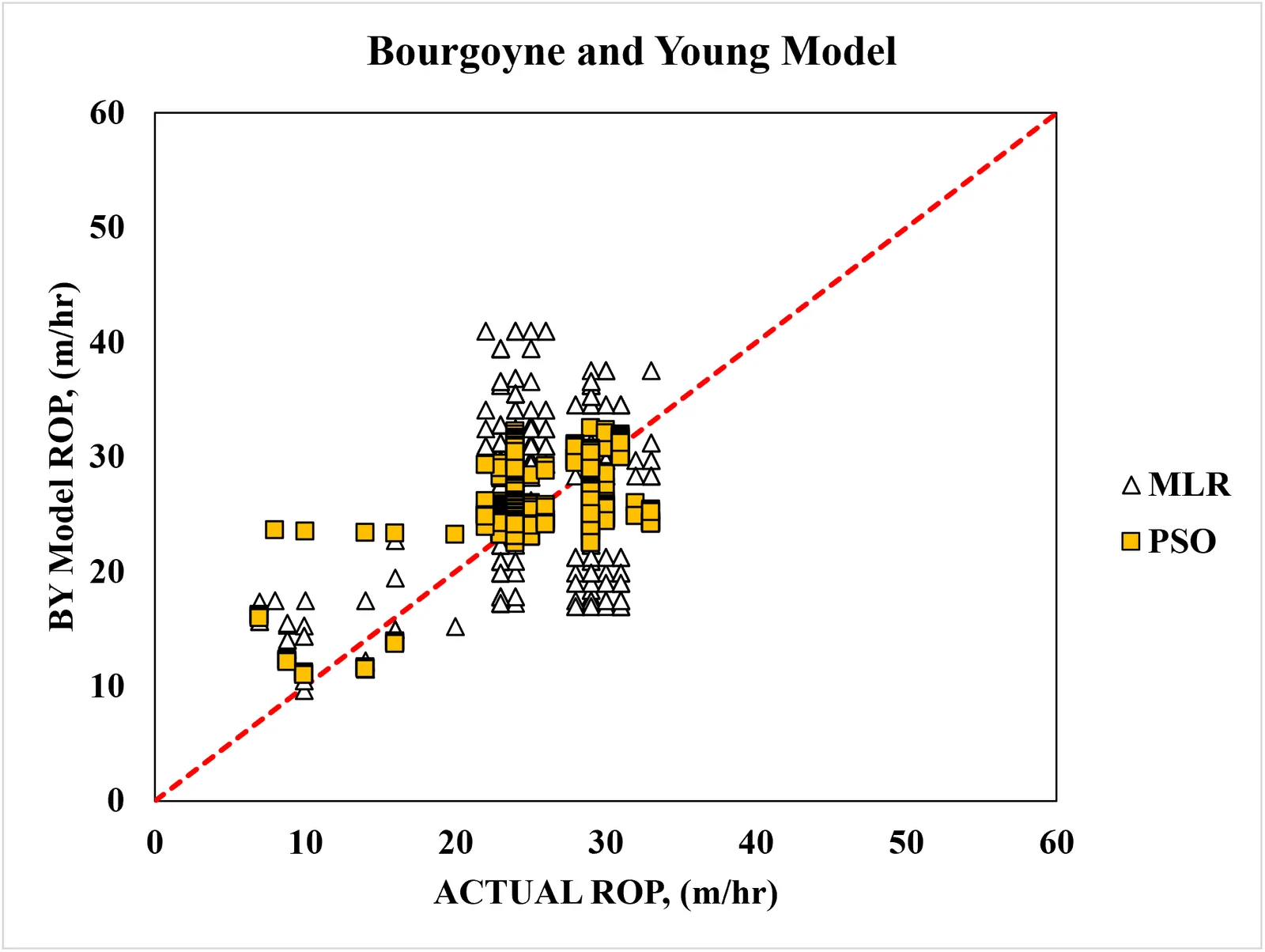
Cross plot of Bourgoyne and Young model. The MLR predictions scatter widely, while the PSO predictions lie closer to the 1:1 line.

**Fig 34:**
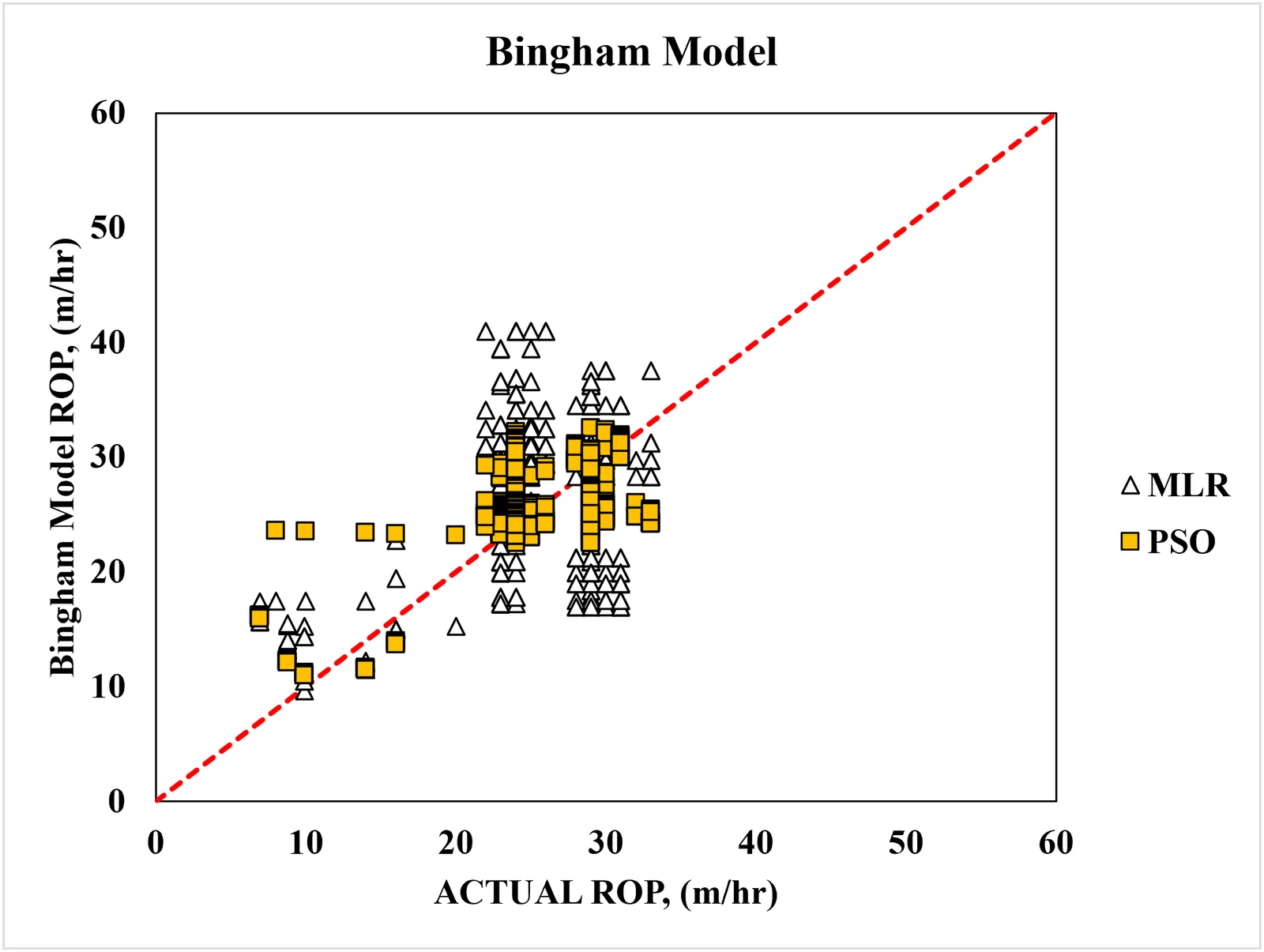
Cross plot of Bingham model. The MLR predictions scatter widely, while the PSO predictions lie closer to the 1:1 line.

From cross plots of empirical models, we can infer that either of the models were not able to predict rate of penetration (ROP) accurately with either multiple regression (MLR) or particle swarm optimization (PSO). The summary results for both techniques are shown in Table 18.

**Table 18:** Performance metrics of empirical models.

Error Metrics	Bourgoyne and Young model		Bingham Model	
	Multiple Regression (MLR)	Particle Swarm Optimization (PSO)	Multiple Regression (MLR)	Particle Swarm Optimization (PSO)
R^2^	0.581	0.414	0.129	0.437
AAPE	11.77%	13.43%	17.56%	11.87%

This high error can be attributed to their nature as a universal model applicable across different field globally. Despite the application of advanced optimization technique to both empirical models, only the Bingham model increased in terms of its correlation coefficient, rising from 0.129 to 0.437. However, the model’s accuracy remains very poor. The two empirical models exhibit lower performance compared to the machine learning models errors substantially surpass those of machine learning models in three scenarios, with both the Bingham model and the Bourgoyne and Young model often demonstrating errors between 20% and 40%, and in particular cases, errors soaring to 80% [30,35,62,63].

### 3.5. Model selection analysis

In the three scenarios analyzed, all machine learning models demonstrated strong prediction accuracy on the testing dataset, with R^2^ > 0.93 and AAPE < 3% across all configurations. Nonetheless, accuracy metrics alone do not ensure physical compliance with recognized principles of drilling mechanics. Analysis trend analysis revealed that RF in scenario 1 was the most physically consistent model, meeting both the WOB flounder point criterion, where ROP increases with WOB to a threshold before leveling off and the rotary speed optimum criterion where ROP ascends with RPM to a maximum before either declining or stabilizing. Table 9 shows the optimum hyperparameters of the RF model under scenario 1, derived via Bayesian optimization to ensure reproducibility.

Figs 35 and 36 illustrate the trend analysis for this model against the empirical models. The WOB flounder point is identified at approximately 13 tons, while the rotary speed optimum point occurs at approximately 132 rpm. Both findings align with the physical expectations of the Bourgoyne and Young drilling model and the Spearman correlation structure of the dataset (r_WOB_ = 0.668 & r_RPM_ = 0.331). The explanations of the statistical criteria are provided in the supporting information.

**Fig 35:**
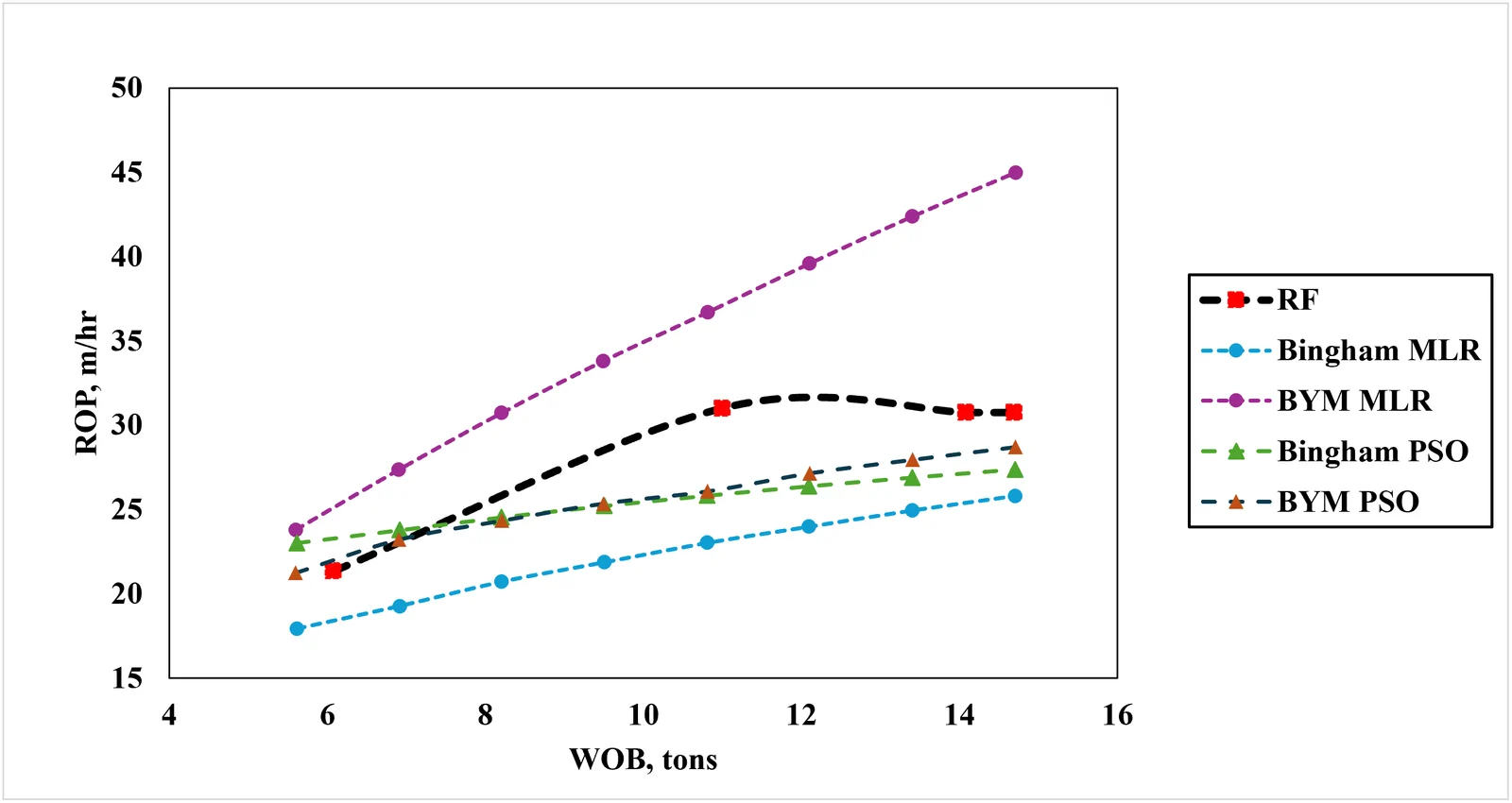
Comparison of the best machine learning model and empirical models for the effect of weight on bit on rate of penetration. RF captures a plateau at about 31 m/hr above roughly 11 tons, whereas the empirical models increase steadily with WOB.

**Fig 36:**
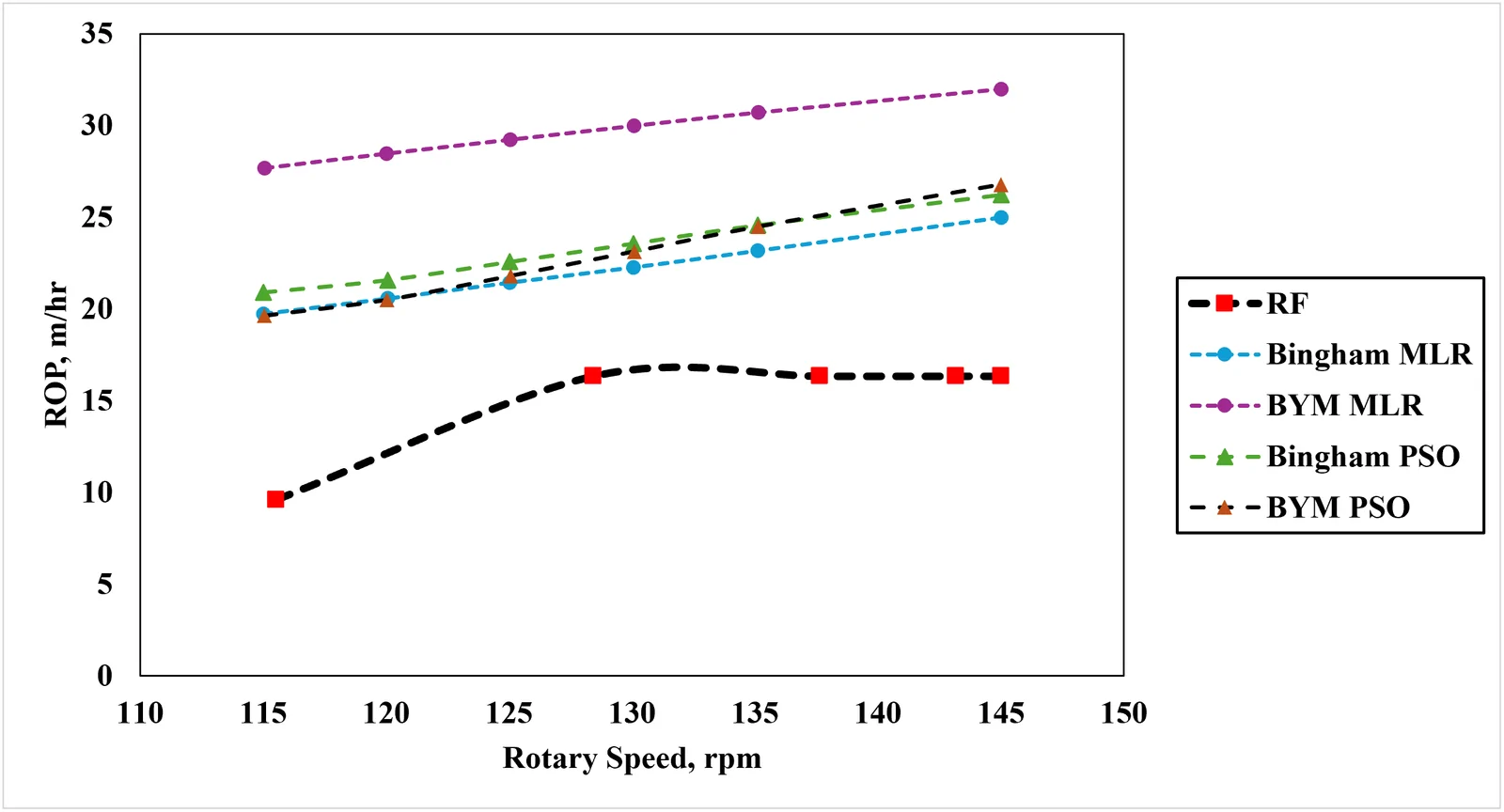
Comparison of the best machine learning model and empirical models for the effect of rotary speed on rate of penetration. RF predicts lower ROP (about 10-17 m/hr) than all four empirical models across the range.

### 3.6. Statistical significance, confidence interval and uncertainty analysis

Three complementary analyses were implemented to evaluate the statistical robustness and reliability of the model comparisons. We used bootstrap resampling [64] to generate 95% confidence intervals around each model’s R^2^ and RMSE for the test-set predictions, indicating the range within which true performance is likely to fall given sampling variability. To ensure that the differences between GBR, RF and KNN that we observed were not due to random variation but rather indicative of real differences in performance, we then applied the Friedman test [65], a non-parametric test that ranks the paired model performance across all test observations. This omnibus test was significant in all three scenarios (p < 1.3*10^−9^). The Friedman test does not specify the different pairs, so post-hoc Wilcoxon signed-rank tests [66] with Holm correction [67] were run for each pair of models on their paired per-row prediction errors. The results are summarized in Table 19.

**Table 19:** Bootstrap 95% confidence intervals, pairwise significance, and conformal prediction coverage.

	Model	R^2^ [95% CI]	Sig. vs GBR (Holm-adj. p)	90% Conformal Coverage	Avg. Interval Width
Scenario 1	GBR	0.986 [0.974, 0.995]	–	95%	2.218
	RF	0.949 [0.885, 0.992]	p = 0.0012 *(significant)*	93.1%	2.323
	KNN	0.936 [0.878, 0.980]	p = 0.305 *(not significant)*	98%	5.543
Scenario 2	GBR	0.979 [0.958, 0.995]	–	94.1	1.969
	RF	0.947 [0.883, 0.991]	p = 0.0002 *(significant)*	93.1	2.148
	KNN	0.962 [0.930, 0.986	p = 0.0032 *(significant)*	98%	4.724
Scenario 3	GBR	0.981 [0.962, 0.995]	–	94.1%	1.764
	RF	0.952 [0.907, 0.987]	p = 0.0001 *(significant)*	92.1%	2.204
	KNN	0.960 [0.928, 0.984]	p = 0.067 *(not significant)*	98%	4.724

Table 19 shows that GBR gave the narrowest confidence intervals and highest point estimates in all scenarios and significantly outperformed RF in all cases. Its superiority over KNN, however, was only statistically significant in scenario 2. Thus, while GBR outperforms KNN in terms of the overall R^2^, the two models are in close agreement on most predictions at the individual level in Scenarios 1 and 3. To directly quantify prediction uncertainty, we also applied split conformal prediction at a 90% target coverage level, using a calibration set held out only from the training part. In this model-agnostic approach the construction guaranties that a certain percentage of the true values will lie in the predicted interval, instead of an ad hoc estimate of the uncertainty. All three models reached or exceeded the 90% target, suggesting that the calibration procedure was successful. GBR had the smallest mean interval widths (1.76–2.22), and KNN had the largest (4.72–5.54), which were approximately 2.4- to 2.7-fold larger than those of GBR, due to its relatively higher predictive uncertainty.

## 4. Model limitations

The models demonstrated a high predictive accuracy and captured trends consistent with physical drilling behavior. However, the generalizability of the models is limited by the geological homogeneity of the dataset. The rate of penetration is essentially governed by the rock mechanical properties such as compressive strength, hardness and degree of consolidation, which vary considerably with lithological type.

The study includes seven drilled formations, which are all shale, sandstone or interbedded shale/sandstone sequences. This lithological uniformity indicates that the models never encountered significantly different rock types, for instance, carbonates, evaporites (e.g., anhydrite salt), or unconsolidated/poorly consolidated formations, all of which have drilling responses significantly different from siliciclastic sequences due to differences in strength, brittleness, and abrasiveness.

Hence, the strong performance reported here should be considered as indicative of ROP behavior in shale–sandstone lithological settings like this well and not as indicative of generalizability to the wider range of lithologies encountered in petroleum drilling operations. Applying the model to wells that significantly penetrate different rock types would probably require retraining or transfer learning with lithology-specific data. The learned relationship between the drilling parameters and ROP is inherently conditioned on the rock mechanics of the formations represented in this dataset.

## 5. Future work recommendations

While this study proves strong predictive performance for GBR, RF, and KNN across three feature scenarios, several possibilities for future work remain.

- The models were constructed and validated on data from a single well in the Muglad Basin. Applying this framework to multiple wells across different formations, or to other basins, would help assess the generalizability of the feature importance rankings and model rankings reported.
- Real-time deployment and online learning approaches can be explored to enable continuous ROP prediction and drilling parameters optimization when new data are available during operations rather than post-drilling analysis.

Future studies can explore hybrid physics and machine learning models that combine the mechanistic basis of Bingham and Bourgoyne-Young formulations with the predictive power of ensemble machine learning approaches that could potentially address the trend-following limitations of the empirical model analysis while maintaining the physical interpretability

## 6. Conclusion

This study developed three machine learning models for rate of penetration (ROP) prediction, and their models were compared to Bingham and Bourgoyne-Young models. The main findings are:

- All three ML models showed good predictive performance (R^2^ > 0.93 & AAPE < 3%) for all feature scenarios on the held-out test data and significantly outperformed the empirical benchmarks, even after optimization.
- High statistical accuracy alone did not guarantee physical consistency, trend analysis versus established drilling relationships showed that only random forest correctly reproduced both physical trends, while GBR, despite superior error metrics performance, displayed a flat, non-physical rotary-speed response in most scenarios and KNN showed boundary instability at extremes of the tested range.
- The random forest in scenario 1 was the most physically consistent model, capturing both the WOB floundering point and rotary speed optimum correctly, while retaining good predictive performance.
- The machine learning models outperformed the empirical models even after optimization, and neither Bingham and Bourgoyne-Young models correctly captured the physical trends of WOB or rotary speed on ROP, reinforcing the value of the machine learning approach for this dataset.

## Supporting information

S1 Table Test-set performance across train:test ratios. (DOCX)

S2 Table Training summary results from all scenarios. (DOCX)

S3 Table Testing summary results from all scenarios. (DOCX)

S1 Fig Cross-plots of GBR model predictions for the training dataset in scenario 1. Predicted versus actual ROP for GBR on the training dataset Scenario 1 with R^2^ = 0.9980 and AAPE = 0.52%.
(TIF)

S2 Fig Cross-plots of KNN model predictions for the training dataset in scenario 1. Predicted versus actual ROP for KNN on the training dataset, Scenario 1 with R^2^ = 0.9603 and AAPE = 1.94%.
(TIF)

S3 Fig Cross-plots of RF model predictions for the training dataset in scenario 1. Predicted versus actual ROP for RF on the training dataset, Scenario 1 with R^2^ = 0.9840 and AAPE = 1.13%.
(TIF)

S4 Fig Cross-plots of GBR model predictions for the training dataset in scenario 2. Predicted versus actual ROP for GBR on the training dataset, Scenario 2 with R^2^ = 0.9959 and AAPE = 0.71%.
(TIF)

S5 Fig Cross-plots of KNN model predictions for the training dataset in scenario 2. Predicted versus actual ROP for KNN on the training dataset, Scenario 2 with R^2^ = 0.9803 and AAPE = 1.16%.
(TIF)

S6 Fig Cross-plots of RF model predictions for the training dataset in scenario 2. Predicted versus actual ROP for RF on the training dataset, Scenario 2 with R^2^ = 0.9821 and AAPE = 1.27%.
(TIF)

S7 Fig Cross-plots of GBR model predictions for the training dataset in scenario 3. Predicted versus actual ROP for GBR on the training dataset, Scenario 3 with R^2^ = 0.9955 and AAPE = 0.74%.
(TIF)

S8 Fig Cross-plots of KNN model predictions for the training dataset in scenario 3. Predicted versus actual ROP for KNN on the training dataset, Scenario 3 with R^2^ = 0.9801 and AAPE = 1.17%.
(TIF)

S9 Fig Cross-plots of RF model predictions for the training dataset in scenario 3. Predicted versus actual ROP for RF on the training dataset, Scenario 3 with R^2^ = 0.9803 and AAPE = 1.46%.
(TIF)

S10 Fig Residual plot of RF model predictions for the testing dataset in scenario 1. The residuals scatter around zero with no clear trend, mostly within ±1 m/hr.
(TIF)

S11 Fig Residuals versus predicted ROP for GBR on the training dataset in scenario 1. The residuals lie tightly around zero, mostly within ±1 m/hr, with no visible trend.
(TIF)

S12 Fig Residuals versus predicted ROP for KNN on the testing dataset in scenario 1. Most residuals fall within about ±2 m/hr, with two larger negative outliers of about −5 and −7 m/hr.
(TIF)

S13 Fig Residuals versus predicted ROP for KNN on the training dataset in scenario 1. The scatter is wider than for GBR, mostly within ±3 m/hr, with larger deviations of about ±5 m/hr at low predicted ROP.
(TIF)

S14 Fig Residuals versus predicted ROP for RF on the testing dataset in scenario 1. Most residuals fall within about ±2 m/hr, with one large negative outlier of about −8 m/hr at low predicted ROP.
(TIF)

S15 Fig Residuals versus predicted ROP for RF on the training dataset in scenario 1. The residuals are mostly within ±3 m/hr, with one larger negative deviation of about −6 m/hr at low predicted ROP.
(TIF)

S16 Fig Residuals versus predicted ROP for GBR on the testing dataset in scenario 2. Most residuals fall within about ±1 m/hr, with one larger negative outlier of about −5 m/hr at low predicted ROP.
(TIF)

S17 Fig Residuals versus predicted ROP for GBR on the training dataset in scenario 2. The residuals are tightly grouped around zero, mostly within ±1.5 m/hr, with no clear trend.
(TIF)

S18 Fig Residuals versus predicted ROP for KNN on the testing dataset in scenario 2. Most residuals fall within about ±2 m/hr, with one negative outlier of about −5 m/hr at low predicted ROP.
(TIF)

S19 Fig Residuals versus predicted ROP for KNN on the training dataset in scenario 2. The scatter is wider than for GBR, mostly within about ±3 m/hr and reaching about ±4 m/hr for a few points.
(TIF)

S20 Fig Residuals versus predicted ROP for RF on the testing dataset in scenario 2. Most residuals fall within about ±2 m/hr, with one large negative outlier of about −8 m/hr at low predicted ROP.
(TIF)

S21 Fig Residuals versus predicted ROP for RF on the training dataset in scenario 2. The residuals are mostly within ±3 m/hr, with one larger negative deviation of about −6 m/hr at low predicted ROP.
(TIF)

S22 Fig Residuals versus predicted ROP for GBR on the testing dataset in scenario 3. Most residuals fall within about ±1 m/hr, with one larger negative outlier of about −4 m/hr at low predicted ROP.
(TIF)

S23 Fig Residuals versus predicted ROP for GBR on the training dataset in scenario 3. The residuals are tightly grouped around zero, mostly within ±1.5 m/hr, with no clear trend.
(TIF)

S24 Fig Residuals versus predicted ROP for KNN on the testing dataset in scenario 3. Most residuals fall within about ±2 m/hr, with one negative outlier of about −5 m/hr at low predicted ROP.
(TIF)

S25 Fig Residuals versus predicted ROP for KNN on the training dataset in scenario 3. The scatter is wider than for GBR, mostly within about ±3 m/hr and reaching about ±4 m/hr for a few points.
(TIF)

S26 Fig Residuals versus predicted ROP for RF on the testing dataset in scenario 3. Most residuals fall within about ±2 m/hr, with one large negative outlier of about −7 m/hr at low predicted ROP.
(TIF)

S27 Fig Residuals versus predicted ROP for RF on the training dataset in scenario 3. The residuals are mostly within ±3 m/hr, with larger deviations of about −5 m/hr and +4 m/hr at low predicted ROP.
(TIF)

### Nomenclature

**Table:** 

AAPE	Average Absolute Percent Error
AARE	Average Absolute Relative Error
ANFIS	Adaptive Neuro-fuzzy Inference System
b	WOB exponent
BY	Bourgoyne and Young Model
CNN	convolutional neural network
D	Maximum Depth
EHTGNN	Explainable Hybrid Temporal Graph Neural Network
d_b_	Bit Diameter
FIS	Fuzzy Inference Systems
GBR	Gradient Boosting Regressor
K	Number of Neighbours
KNN	K-Nearest Neighbours
LSTM	Long Short-Term Memory
MAE	Mean Absolute Error
MLR	Multiple Regression
MAPE	Mean Absolute Percentage Error
MW	Mud Weight
PSO	Particle Swarm Optimization
r	Spearman Rank Correlation Coefficient
R^2^	Correlation of Determination
RF	Random Forest
RMSE	Root Mean Squared Error
ROP	Rate of Penetration
RPM	Revolutions Per Minute
SPP	Standpipe Pressure
VIF	Variance Inflation Factor
WOB	Weight on Bit
σ	Standard Deviation
µ	Mean
