# Supplementary material for: Predicting rate of penetration (ROP) with machine learning models: A physics-validated comparative case study of Gradient Boosting Regressor (GBR), Random Forest (RF), and K-Nearest Neighbours (KNN)
Source: PLoS One. 2026 Nov 6;21(11):e0360046. doi: 10.1371/journal.pone.0360046 (PMC13742925; doi:10.1371/journal.pone.0360046)
Supplement: Supplementary file 1 [file pone.0360046.s001.docx]

| **Feature Scenario** | **Model** | **Train:Test Ratio** | **Test R² (mean ± SD)** | **Test RMSE (mean ± SD)** |
| --- | --- | --- | --- | --- |
| All 6 Features | GBR | 60:40 | 0.957 ± 0.015 | 0.918 ± 0.192 |
| All 6 Features | GBR | 70:30 | 0.969 ± 0.010 | 0.768 ± 0.107 |
| All 6 Features | GBR | 80:20 | 0.980 ± 0.008 | 0.606 ± 0.141 |
| All 6 Features | GBR | 85:15 | 0.978 ± 0.010 | 0.633 ± 0.202 |
| All 6 Features | RF | 60:40 | 0.948 ± 0.024 | 1.003 ± 0.246 |
| All 6 Features | RF | 70:30 | 0.959 ± 0.013 | 0.896 ± 0.154 |
| All 6 Features | RF | 80:20 | 0.973 ± 0.013 | 0.697 ± 0.208 |
| All 6 Features | RF | 85:15 | 0.975 ± 0.014 | 0.671 ± 0.255 |
| All 6 Features | KNN | 60:40 | 0.871 ± 0.032 | 1.602 ± 0.214 |
| All 6 Features | KNN | 70:30 | 0.903 ± 0.028 | 1.368 ± 0.200 |
| All 6 Features | KNN | 80:20 | 0.925 ± 0.016 | 1.187 ± 0.136 |
| All 6 Features | KNN | 85:15 | 0.925 ± 0.017 | 1.177 ± 0.152 |
| Torque Removed | GBR | 60:40 | 0.958 ± 0.015 | 0.909 ± 0.192 |
| Torque Removed | GBR | 70:30 | 0.969 ± 0.006 | 0.776 ± 0.093 |
| Torque Removed | GBR | 80:20 | 0.978 ± 0.006 | 0.646 ± 0.123 |
| Torque Removed | GBR | 85:15 | 0.977 ± 0.011 | 0.657 ± 0.211 |
| Torque Removed | RF | 60:40 | 0.941 ± 0.028 | 1.068 ± 0.261 |
| Torque Removed | RF | 70:30 | 0.953 ± 0.017 | 0.955 ± 0.173 |
| Torque Removed | RF | 80:20 | 0.966 ± 0.014 | 0.787 ± 0.208 |
| Torque Removed | RF | 85:15 | 0.970 ± 0.015 | 0.747 ± 0.260 |
| Torque Removed | KNN | 60:40 | 0.907 ± 0.020 | 1.366 ± 0.199 |
| Torque Removed | KNN | 70:30 | 0.927 ± 0.019 | 1.190 ± 0.162 |
| Torque Removed | KNN | 80:20 | 0.941 ± 0.011 | 1.055 ± 0.138 |
| Torque Removed | KNN | 85:15 | 0.937 ± 0.012 | 1.086 ± 0.159 |
| MW_avg & Torque Removed | GBR | 60:40 | 0.955 ± 0.026 | 0.925 ± 0.227 |
| MW_avg & Torque Removed | GBR | 70:30 | 0.971 ± 0.007 | 0.747 ± 0.109 |
| MW_avg & Torque Removed | GBR | 80:20 | 0.982 ± 0.006 | 0.583 ± 0.121 |
| MW_avg & Torque Removed | GBR | 85:15 | 0.984 ± 0.007 | 0.552 ± 0.144 |
| MW_avg & Torque Removed | RF | 60:40 | 0.941 ± 0.031 | 1.061 ± 0.250 |
| MW_avg & Torque Removed | RF | 70:30 | 0.953 ± 0.015 | 0.950 ± 0.160 |
| MW_avg & Torque Removed | RF | 80:20 | 0.968 ± 0.012 | 0.772 ± 0.178 |
| MW_avg & Torque Removed | RF | 85:15 | 0.973 ± 0.010 | 0.716 ± 0.209 |
| MW_avg & Torque Removed | KNN | 60:40 | 0.908 ± 0.021 | 1.359 ± 0.201 |
| MW_avg & Torque Removed | KNN | 70:30 | 0.928 ± 0.020 | 1.176 ± 0.182 |
| MW_avg & Torque Removed | KNN | 80:20 | 0.943 ± 0.012 | 1.032 ± 0.135 |
| MW_avg & Torque Removed | KNN | 85:15 | 0.943 ± 0.013 | 1.035 ± 0.160 |
