# Supplementary material for: Predicting rate of penetration (ROP) with machine learning models: A physics-validated comparative case study of Gradient Boosting Regressor (GBR), Random Forest (RF), and K-Nearest Neighbours (KNN)
Source: PLoS One. 2026 Nov 6;21(11):e0360046. doi: 10.1371/journal.pone.0360046 (PMC13742925; doi:10.1371/journal.pone.0360046)
Supplement: Supplementary file 2 [file pone.0360046.s002.docx]

| **Training** | | | | | | | | | |
| --- | --- | --- | --- | --- | --- | --- | --- | --- | --- |
| **Models** | **Scenario 1** | | | **Scenario 2** | | | **Scenario 3** | | |
|  | GBR | KNN | RF | GBR | KNN | RF | GBR | KNN | RF |
| **R^2^** | 0.998 | 0.960 | 0.984 | 0.996 | 0.980 | 0.982 | 0.995 | 0.980 | 0.980 |
| **RMSE** | 0.209 | 0.934 | 0.592 | 0.300 | 0.659 | 0.626 | 0.316 | 0.661 | 0.659 |
| **MAE** | 0.111 | 0.494 | 0.214 | 0.141 | 0.292 | 0.230 | 0.153 | 0.294 | 0.243 |
| **AAPE** | 0.52% | 1.94% | 1.13% | 0.71% | 1.16% | 1.27% | 0.744% | 1.17% | 1.46% |
