# Supplementary material for: Predicting rate of penetration (ROP) with machine learning models: A physics-validated comparative case study of Gradient Boosting Regressor (GBR), Random Forest (RF), and K-Nearest Neighbours (KNN)
Source: PLoS One. 2026 Nov 6;21(11):e0360046. doi: 10.1371/journal.pone.0360046 (PMC13742925; doi:10.1371/journal.pone.0360046)
Supplement: Supplementary file 3 [file pone.0360046.s003.docx]

| **Testing** | | | | | | | | | |
| --- | --- | --- | --- | --- | --- | --- | --- | --- | --- |
| **Models** | **Scenario 1** | | | **Scenario 2** | | | **Scenario 3** | | |
|  | GBR | KNN | RF | GBR | KNN | RF | GBR | KNN | RF |
| **R^2^** | 0.986 | 0.936 | 0.949 | 0.978 | 0.962 | 0.947 | 0.981 | 0.960 | 0.952 |
| **RMSE** | 0.505 | 1.070 | 0.954 | 0.617 | 0.831 | 0.976 | 0.591 | 0.851 | 0.927 |
| **MAE** | 0.213 | 0.433 | 0.270 | 0.237 | 0.294 | 0.271 | 0.242 | 0.330 | 0.280 |
| **AAPE** | 1.17% | 2.25% | 1.96% | 1.45% | 1.55% | 2.02% | 1.42% | 1.68% | 2.06% |
